# Dynamin-related protein 2 interacts with the membrane-associated methyltransferase domain of plantago asiatica mosaic virus replicase and promotes viral replication

**DOI:** 10.1016/j.virusres.2023.199128

**Published:** 2023-05-14

**Authors:** Haruka Shinji, Nobumitsu Sasaki, Islam Hamim, Yoshiyuki Itoh, Kazuo Taku, Yuho Hayashi, Nami Minato, Hiromitsu Moriyama, Tsutomu Arie, Ken Komatsu

**Affiliations:** a Graduate School for Agriculture, Tokyo University of Agriculture and Technology (TUAT), Tokyo 183-8509, Japan; b Virology, Translational Plant Pathology and Agrosecurity Laboratory, Department of Plant Pathology, Bangladesh Agricultural University, Mymensingh-2202, Bangladesh; c International Research Fellow, Japan Society for the Promotion of Science, Tokyo 102-0083, Japan; d Smart-Core-Facility Promotion Organization, Tokyo University of Agriculture and Technology (TUAT), Tokyo 183-8509, Japan; e Institute of Science and Technology, Niigata University, Niigata 950-2181 Japan

**Keywords:** Plantago asiatica mosaic virus, *Nicotiana benthamiana*, *Arabidopsis thaliana*, IP-MS analysis, Methyltransferase domain, Viral replication complex

## Abstract

Positive-strand RNA viruses replicate their RNA in the viral replication complex, a spherical structure formed by remodeling of host intracellular membranes. This process also requires the interaction between viral membrane-associated replication proteins and host factors. We previously identified the membrane-associated determinant of the replicase of plantago asiatica mosaic virus (PlAMV), a positive-strand RNA virus of the genus *Potexvirus*, in its methyltransferase (MET) domain, and suggested that its interaction with host factors is required to establish viral replication. Here we identified *Nicotiana benthamiana* dynamin-related protein 2 (NbDRP2) as an interactor of the MET domain of the PlAMV replicase by co-immunoprecipitation (Co-IP) and mass spectrometry analysis. NbDRP2 is closely related to the DRP2 subfamily proteins in *Arabidopsis thaliana*, AtDRP2A and AtDRP2B. Confocal microscopy observation and Co-IP confirmed the interaction between the MET domain and NbDRP2. Also, the expression of *NbDRP2* was induced by PlAMV infection. PlAMV accumulation was reduced when the expression of *NbDRP2* gene was suppressed by virus-induced gene silencing. In addition, PlAMV accumulation was reduced in protoplasts treated with dynamin inhibitor. These results indicate a proviral role of the interaction of NbDRP2 with the MET domain in PlAMV replication.

## Highlights

• The MET domain of PlAMV replicase interacts with NbDRP2 in *Nicotiana benthamiana*.
• PlAMV infection induced *NbDRP2* gene expression.
• The accumulation level of PlAMV was decreased in the *NbDRP2* knockdown plants.
• PlAMV accumulation was suppressed in protoplasts treated with dynamin inhibitor.
• NbDRP2 plays a proviral role during PlAMV replication.

## 1 Introduction

Positive-sense, single-stranded RNA ((+)ssRNA) viruses infect a wide range of plants and animals and cause significant economic losses. Upon entry into a host cell, viral genomic RNA is released from the viral particle and translated by host ribosomes to synthesize viral proteins necessary for replication, such as RNA-dependent RNA polymerase (RdRp, replicase). The replicase of (+)ssRNA viruses uses viral genomic RNA as a template to produce complementary negative-strand RNA, which is then used as a template to produce a large amount of positive-strand RNA (Ahlquist et al., 2003). This viral replication process occurs in membrane-bound spherules called viral replication complexes (VRCs), which are formed by modification of the host intracellular membranes, including the endoplasmic reticulum (ER), mitochondria, and chloroplasts. VRCs contain viral genomic RNA, replicase, and other viral proteins, as well as host proteins and lipids that play vital roles in viral replication by generating a suitable environment for viral replication and protecting viral RNA from host antiviral responses (Laliberte'and Zheng, 2014; Kovalev et al., 2017). Following replicase binding to membranes, the assembly of viral replication enzymes and replication auxiliary proteins is crucial for VRC formation. For example, cucumber necrosis virus (CNV) forms VRCs on the peroxisomal membrane through the self-assembly of p33, a replication auxiliary protein, and its binding to p92^pol^ protein, which has polymerase activity (Panavas et al., 2005). VRC formation in red clover necrotic mosaic virus (RCNMV) also requires the self-assembly of p27, a replication-associated protein, and its interaction with p88 polymerase (Kusumanegara et al., 2012; Mine et al., 2010a). Recent studies have shown that the replicases of some (+)ssRNA viruses assemble into the symmetric “crown” complex at the spherule neck, which is important for VRC formation (Nishikiori et al., 2022). In addition to the assembly of viral replication proteins, VRC formation and viral replication also require interactions between viral proteins and host factors. Tomato bushy stunt virus (TBSV) replication proteins p33 and p92^pol^ interact with proteins involved in the regulation of lipid transport and vesicle trafficking to create the membrane microenvironment suitable for viral replication (Kovalev et al., 2016; McCartney et al., 2005; Xu and Nagy, 2017; Barajas et al., 2014). Another study has shown that host factors involved in intracellular vesicle trafficking are important for VRC formation. For example, p27 of RCNMV interacts with ADP ribosylation factor 1 (Arf1), which regulates the synthesis of coatomer protein I (COPI) involved in ER-Golgi retrograde trafficking, to recruit Arf1 from the Golgi to the VRC (Hyodo and Okuno, 2016). It has been suggested that RCNMV VRC contains multiple host factors in addition to viral RNA and viral replication auxiliary proteins (Mine et al., 2010b). Therefore, in addition to viral protein assembly, interactions with host factors are generally critical for supporting viral replication (Wang, 2015; Nagy et al., 2012).

Plantago asiatica mosaic virus (PlAMV) is a (+)ssRNA virus in the genus *Potexvirus* of the family *Alphaflexiviridae* of the phylum *Tymovirales*. The PlAMV genome has five open reading frames (ORFs), of which ORF1 encodes replicase (Komatsu et al., 2011), ORFs 2, 3, and 4 the movement proteins triple gene block proteins 1, 2, and 3 (TGBp1, TGBp2, and TGBp3), respectively, and ORF5 a coat protein (Komatsu and Hammond, 2022). An advantage of using PlAMV as a model plant virus is the availability of a green fluorescent protein (GFP) expression vector that efficiently infects the model plant *Arabidopsis thaliana*, contributing to the identification of several host factors that inhibit or facilitate virus replication (Yamaji et al., 2012; Hashimoto et al., 2016). It has also been reported that PlAMV replication is inhibited by the plant activator acibenzolar-S-methyl (Matsuo et al., 2019). However, the detailed mechanisms by which these host factors and the plant activator are involved in PlAMV replication remain unknown. Further understanding of the function of host factors involved in viral replication is needed to elucidate these mechanisms.

The replicase of the PlAMV is composed of the methyltransferase (MET) domain, helicase (HEL) domain, and RNA polymerase (POL) domain. Recently, the MET domain with a proline (P369)-kinked amphipathic α-helix located at its C-terminal region was identified as the membrane-associated domain (Komatsu et al., 2021). The MET domain forms small punctate structures and a large perinuclear complex in close association with ER membranes when transiently expressed in plants. In particular, by mutational analysis of this helix, we found that several single mutations in the MET domain caused a loss of its ability to form the large complex and that PlAMV variants with these mutation in their MET domain were replication defective. On the other hand, we also discovered a MET-P369L mutant that caused PlAMV to lose its ability to replicate while retaining its ability to form large complexes. This suggests that the large complex-forming ability of the MET domain is essential but not sufficient for virus replication; the MET domain may have other functions in establishing virus replication. Given the numerous previous studies demonstrating that interactions between viral replication auxiliary proteins and host factors are critical for viral replication within the VRC, it is hypothesized that the MET domain interacts with some host factors to utilize their function for virus replication.

Dynamin is a widely conserved membrane protein in eukaryotes and is involved in the regulation of membrane structure and intracellular trafficking. It was first identified in animal cells (Shpetner and Vallee, 1989), where it was found to be required for the formation of clathrin-coated vesicles during endocytosis (Takei et al., 1995; Sever, 2002). Besides that, dynamin-related proteins (DRPs) are found in plants. In *A. thaliana*, these DRPs are classified into six subfamilies (DRP1–6) depending on their amino acid sequence and domain structures (Hong et al., 2003). The N-terminal dynamin GTPase domain, the middle domain that facilitates dynamin self-assembly, and the GTPase effector domain that modulates GTPase activity, are conserved in all subfamilies. In addition to these three domains, DRP2A and DRP2B in *A. thaliana* contain a Pleckstrin homology (PH) domain, which is involved in membrane curvature, and a proline-rich domain (Fujimoto et al., 2010). Members of the *A. thaliana* DRP1 subfamily localize to the cell plate and are involved in membrane remodeling, vesicle formation, and cell plate formation (Fujimoto et al., 2010). *A. thaliana* DRP2A (AtDRP2A) and DRP2B (AtDRP2B) genes are constitutively expressed and have 93% amino acid similarity (Bednarek and Backues, 2010). *A. thaliana* DRP2s localize to the plasma membrane, cell plate, and post-Golgi organelles and are involved in vesicle trafficking, cell plate formation, and immune responses to bacterial infection (Fujimoto et al., 2010; Konopka and Bednarek, 2008; Smith et al., 2014). Furthermore, AtDRP2A and AtDRP2B have redundant functions; single mutants of these genes show normal growth, whereas their double mutant shows significant growth defects (Backues et al., 2010). Moreover, a recent study has shown that some DRPs function in promoting potyvirus infection. DRP2A and DRP2B in *A. thaliana* promote turnip mosaic virus (TuMV) replication, and the soybean DRP1 homologs dynamin-like protein 5A (GmSDL5A) and 12A (GmSDL12A) facilitate soybean mosaic virus (SMV) replication (Wu et al., 2018). However, it is not clear whether dynamin is involved in infection of other group of plant viruses.

In this study, we identified host factors that interact with the MET domain in *Nicotiana benthamiana* by co-immunoprecipitation and mass spectrometry. We focused on dynamin-related protein 2 (NbDRP2), which was shown to interact not only with MET-Wt but also with the mutants MET-P369L and MET-L363A by co-immunoprecipitation. Moreover, we observed the colocalization of the MET domain and NbDRP2 in plant cell by confocal microscopy observation. We also found that PlAMV utilizes NbDRP2 to promote viral replication. Overall, we demonstrated that NbDRP2 plays a proviral role in PlAMV replication.

## 2 Materials and methods

### 2.1 Plants and viruses, virus inoculation, and agroinfiltration

*Nicotiana benthamiana* plants were grown in a growth chamber under 16 h light/8 h dark conditions at 25°C. Col-0 and its mutants (*atdrp2a* (SALK_071036C) and *atdrp2b* (SALK_134887C)), obtained from the Arabidopsis Biological Resource Center, were grown in a growth chamber under 16 h light/8 h dark conditions at 21°C. Genotyping PCR was performed using the primer set of SALK_071036_LP and SALK_071036_RP for *atdrp2a*, and the primer set of SALK_134887_LP and SALK_134887_RP for *atdrp2b* (Supplementary Table 2). Virions of Li1 isolate of plantago asiatica mosaic virus (PlAMV) were purified as previously described in Matsuo et al. (2019). An infectious clone of Li1 isolate was described previously (Ozeki et al., 2006), which is used for virus inoculation by agroinfiltration. TocJ/GFP, a GFP-expressing tomato mosaic virus (ToMV) (Hori and Watanabe, 2003), and Y strain of cucumber mosaic virus (CMV-Y) propagated in *N. benthamiana* plants were used as an inoculum. Agroinfiltration was performed as previously described (Senshu et al., 2009) for PlAMV infection, the transient expression of viral proteins and virus-induced gene silencing (VIGS) system with the TRV vectors, and transient knockdown using hairpin constructs. Mechanical inoculation of Li1 and PlAMV-GFP (Minato et al., 2014) virions using carborundum was performed as described previously (Matsuo et al., 2019). For VIGS, three- to four-week-old *N. benthamiana* was agroinfiltrated with a mixture of Agrobacterium containing pBINTRA6 and that containing pTV-00 (empty vector) (Shi et al., 2021), or pTV-NbDRP2.

### 2.2 Plasmid construction

The *NbDRP2* gene of *N. benthamiana* was amplified by RT-PCR using its total RNA as a template using a primer set attB1_NbDRP2_F and attB2_NbDRP2_R with the PrimeScript II High Fidelity One Step RT-PCR Kit (Takara bio, Shiga, Japan). The amplified fragment was cloned into the entry vector pDONR/Zeo (Thermo Fisher Scientific, Massachusetts, USA) to create pDONR/Zeo-NbDRP2 after the addition of attB1/B2 sequences and BP reaction using Gateway BP Clonase Enzyme mix (Thermo Fisher Scientific), as described previously (Sasaki et al., 2018).

To construct the plasmid used for the analysis of the interaction between the MET domain and NbDRP2, wild-type or mutated MET fragment was amplified by PCR using the primer set attB1_MET_F and attB2_MET_R by the KOD-Plus-Neo (Toyobo, Osaka, Japan) using pLi1 (Ozeki et al., 2006), MET-P369L-GFP, and MET-L363A-GFP (Komatsu et al., 2021) as a template. The amplified fragment was cloned into pDONR/Zeo by BP reaction to create pDONR/Zeo-MET-Wt, pDONR/Zeo-MET-P369L and pDONR/Zeo-MET-L363A. pEarleyGate-MET-Wt-Cmyc, pEarleyGate-MET-P369L-Cmyc and pEarleyGate-MET-L363A-Cmyc were produced by LR reaction with pEarleyGate-Cmyc (Okano et al., 2014). RT-PCR and BP/LR reactions were performed according to the manufacturer's instructions. NbDRP2 gene was amplified with PCR by the KOD-Plus-Neo (Toyobo) using a primer set GFP_NbDRP2_F and GFP_NbDRP2_R using pDONR/Zeo-NbDRP2 as template to add overlapping sequences for In-Fusion cloning. The amplified fragment was inserted into pCAMBIA1301.1-sGFP (Komatsu et al., 2021) digested with BamHI and KpnI using In-Fusion Snap Assembly Master Mix (Takara bio) to generate pCAMBIA-sGFP-NbDRP2. To construct MET-mCherry, pCAMBIA1301.1-mCherry was constructed by cloning the mCherry gene into pCAMBIA1301 using the same method as that used to construct pCAMBIA1301.1-sGFP shown in Komatsu et al. (2021). The full-length fragment of the MET domain was amplified by PCR using T7-Li1-GFP as a template and a primer set Li-MD-F and Li-MD-R. The amplified fragment was digested with SalI and SpeI and ligated into pCAMBIA1301.1-mCherry, which was digested with the same enzymes.

To construct a plasmid used to knockdown of NbDRP2 gene in *N. benthamiana*, the target sequence for *NbDRP2* gene was designed using pssRNAit (https://www.zhaolab.org/pssRNAit/) (Supplementary Fig. 2). The target fragment was amplified by RT-PCR using *N. benthamiana* total RNA as a template and a primer set pTV_NbDRP2_F and pTV_NbDRP2_R to add overlapping sequences for In-Fusion cloning. The amplified fragment was inserted into pTV-00 digested with SpeI and KpnI using In-Fusion Snap Assembly Master Mix (Takara bio) to generate pTV-NbDRP2. All constructed plasmids were confirmed by restriction enzyme treatment and Sanger sequencing analysis (Eurofins Genomics, Tokyo, Japan). All primers used for plasmid construction are shown in Supplementary Table 2.

To construct a plasmid used for transient knockdown of *NbDRP2* genes in *N. benthamiana*, the target fragment was amplified with PCR using a primer set NbDRP2-attB1–1F and NbDRP2-attB2–199R with pTV-NbDRP2 as a template. The amplified fragment was cloned into pDONR/Zeo by BP reaction. The resulting plasmid was cloned into pBI-sense, antisense-GW (Nemoto et al., 2009) by LR reaction to create pBI-sas-NbDRP2. Control construct pBI-sas-mGFP5 was constructed similarly using a primer set mGFP5-attB1–1F and mGFP5-attB2–400R with mGFP5 sequence as a template (Netsu et al., 2015).

### 2.3 Co-immunoprecipitation and SDS-PAGE

Three- to four-week-old *N. benthamiana* leaves were agroinfiltrated with Agrobacterium expressing MET-Wt-GFP, MET-P369L-GFP, or MET-L363A-GFP (Komatsu et al., 2021). Approximately 0.3 g of agroinfiltrated leaves were collected 24 h post infiltration and ground with 1 mL of buffer A (50 mM HEPES-NaOH pH 7.5, 150 mM NaCl, 5% glycerol, 0.5% Triton X-100, 0.1% (v/v) 3-mercapto-1, 2-propanediol, 1 tablet of cOmplete Mini protease inhibitor cocktail (Roche, Basel, Switzerland) per 10 mL). The resulting solution was centrifuged twice at 1000 x *g*, 4°C for 10 min to obtain crude protein extract. The crude protein was mixed with an equal volume of 2×SDS-PAGE sample buffer (Laemmli sample buffer (BIO-RAD, California, USA) 950 µl/mL, 3-mercapto-1, 2-propanediol 50 µl/mL), and denatured at 95°C for 5 min and stored at −30°C before use.

For immunoprecipitation, the crude protein extracts were incubated with GFP-trap agarose beads (Chromotek, Planegg, Germany) for binding in a cold room for 1 h for immunoprecipitation and 3 h for mass spectrometry. After incubation, the beads were washed 4–6 times with buffer A and proteins were eluted with 1×SDS-PAGE sample buffer (50% 2×SDS-PAGE sample buffer, 50% 1×PBS). Proteins obtained by co-immunoprecipitation were separated by SDS-PAGE using a 12.5% SDS-PAGE gel (e-PAGEL) (ATTO, Tokyo, Japan). The gel was stained with SimplyBlue Safe Stain (Thermo Fisher Scientific), and protein bands of interest were excised with a scalpel.

### 2.4 Western blotting

Proteins separated by SDS-PAGE were transferred to a polyvinylidene difluoride (PVDF) membrane (Merck Millipore, Massachusetts, USA) and detected using the iBind Western Device (Thermo Fisher Scientific) with the following primary antibodies: anti-GFP antibody clones 7.1 and 13. 1 (1:1333, #11814460001, Roche), anti-myc antibody clone 4A6 (1:1143, #05–724, Merck Millipore). Peroxidase-conjugated goat anti-mouse IgG (1:4000, #32430 Thermo Fisher Scientific) was used as a secondary antibody. Signals were detected using ImmunoStar LD substrates (Fujifilm Wako pure chemical co., Osaka, Japan), and images were captured using LAS-3000 (Fujifilm, Tokyo, Japan).

### 2.5 In-gel digestion of proteins and mass spectrometry

Each of the excised gels after SDS-PAGE was decolorized by washing three times with 100 µl of decolorizing solution 1 (25 mM ammonium bicarbonate, 30% acetonitrile) and then 100 µl of decolorizing solution 2 (25 mM ammonium bicarbonate, 50% acetonitrile). After replacing decolorizing solution 2 with 100 µl of acetonitrile, the gel slices were vortexed for 10 min. After removing the acetonitrile, the gel slices were dried by centrifugation under vacuum using a centrifugal evaporator CVE-2000 (EYELA, Tokyo, Japan). Proteins were reduced in 100 µl of reducing solution (10 mM dithiothreitol, 49.5 mM ammonium bicarbonate) and incubated at 37°C, 230 rpm for 60 min. 100 µl of 25 mM ammonium bicarbonate was added to the gel slices, which were then vortexed for 10 min at room temperature. Proteins were alkylated in 100 µl of alkylation solution (46 mM iodoacetamide prepared by dilution with 25 mM ammonium bicarbonate) and incubated in the dark for 45 min at room temperature with continuous shaking. After removal of the solution, the gel slices were washed in 100 µl of 25 mM ammonium bicarbonate and 400 µl of decolorizing solution 2 and then dried by vacuum centrifugation.

Proteins were digested in 20 µg/mL trypsin solution at 37°C with continuous shaking overnight. Peptides were extracted from the gel slices by sonication in 50 µl of extraction solution 1 (50% acetonitrile, 0.1% formic acid), extraction solution 2 (100% acetonitrile, 0.1% formic acid), and extraction solution 3 (0.1% formic acid) for 10 min at room temperature after the addition of each extraction solution. Peptide extracts were concentrated to approximately 75 µl and stored at −80°C until LC-MS/MS analysis using an LTQ-Orbitrap XL (Thermo Fisher Scientific). The resulting mass spectral data were searched in the *N. benthamiana* protein database (Kourelis et al., 2019) using Mascot Server (Matrix Science Inc., Boston, MA, USA) for protein identification.

### 2.6 Phylogenetic analysis of dynamin genes

Homologs of NbDRP2 gene were identified by BLAST searches in the Solgenomics network (https://solgenomics.net/). A phylogenetic tree was constructed based on the predicted full-length amino acid sequences of NbDRP2 and its related homologs, as well as dynamin proteins from *Glycine max, A. thaliana*, and *N. tabacum*. Amino acid sequence data of Arabidopsis dynamin-related proteins were obtained from Uniprot (https://www.uniprot.org/) and the Arabidopsis Information Resource (https://www.arabidopsis.org/index.jsp). Amino acid sequence data of GmSDL5A and GmSDL12A and those of *N. tabacum* dynamin proteins were obtained from Soy Base (https://soybase.org/) and the National Center for Biological Information (https://www.ncbi.nlm.nih.gov/), respectively. Maximum likelihood phylogenetic trees (LG+G+I model, bootstrap replication value 1000) were generated using MEGA7 (Kumar et al., 2016; Le and Gascuel, 2008; Tamura et al., 2021). The accession numbers for each protein are shown in Supplementary Table 1. The amino acid sequences deduced from the full-length ORF of *NbDRP2, AtDRP2A*, and *AtDRP2B* were aligned using MUSCLE in MEGAX (Edgar, 2004). The domain structures of these proteins were obtained using InterProScan (Paysan-Lafosse et al., 2023).

### 2.7 Treatment of protoplasts with chemical inhibitors and virus inoculation

Preparation of *N. benthamiana* protoplasts and inoculation with transcribed viral RNA were performed as described previously (Komatsu et al., 2021). To the protoplast suspension, 10 mM Dynasore hydrate (Sigma-Aldrich) diluted in dimethyl sulfoxide (DMSO) was added so that the final concentration of the Dynasore hydrate solution was 100 µM. DMSO was used for control experiments. Virus inoculation was performed with 2 µg of Li1-GFP transcribed RNA by the polyethylene glycol method. After virus inoculation, 5 µl (100 µM final concentration) of the Dynasore hydrate solution was added to the protoplasts. GFP fluorescence observation and RNA extraction were performed after 24 and 36 h of incubation at 25°C, respectively. Viability of protoplast after inhibitor treatment was confirmed by fluorescein diacetate staining (Fujifilm Wako pure chemical co.).

### 2.8 RNA extraction and reverse transcription quantitative PCR (RT-qPCR)

RNA extraction from plants, cDNA synthesis, and RT-qPCR were performed essentially as described previously (Komatsu et al., 2021). RNA extraction was performed with ISOGEN (Nippon gene, Japan) according to the manufacturer's instructions. 2 µg of total RNA was treated with RQ1 RNase-Free DNase (Promega, Wisconsin, USA), and the DNase-treated RNA was used as a template for cDNA synthesis with ReverTra Ace (Toyobo) according to the manufacturer's protocol. For RT-qPCR to analyze virus accumulation in *N. benthamiana* protoplasts, 50 ng of total RNA was used for cDNA synthesis with SuperScript IV Reverse Transcriptase (Thermo Fisher Scientific), and virus accumulation was quantified by an absolute quantification method using a primer set PlAMV3877F and PlAMV4010R (Tanaka et al., 2019).

Virus accumulation and NbDRP2 gene expression in plants were relatively quantified by normalization to the reference gene *NbPP2A* using a primer set NbPP2A_F and NbPP2A_R (Zhang et al., 2019). A primer set 09472_F and 09472_R, designed based on Niben101Scf09472g02009.1, and a primer set PlAMV3877F and PlAMV4010R, was used for the quantification of the expression of *NbDRP2* gene, and the accumulation of PlAMV-GFP, respectively. A primer set TogJ_F08 and TogJ_R09 and a primer set CMV_RNA1_F and CMV_RNA1_R was used for the quantification of the accumulation of TocJ/GFP and CMV, respectively. GoTaq qPCR Master Mix (Promega) was used for qPCR, and the primer sequences are shown in Supplementary Table 2.

### 2.9 Fluorescence observation of virus infection

The fluorescent spots of PlAMV-GFP infection in *N. benthamiana* plants were visualized with a hand-held UV lamp (UVGL-58; Funakoshi, Japan) in a dark room and photographed with a digital camera EOS90D (Canon, Tokyo). PlAMV-GFP infection in protoplasts was observed using a BX53 microscope (Olympus, Tokyo, Japan) and a U-HGLPS light source (Olympus) as described previously (Saito et al., 2021), and virus infection in *A. thaliana* was observed as described previously (Matsuo et al., 2019). The number of fluorescent spots in the observed images was quantified using ImageJ software (https://imagej.nih.gov/ij/download.html).

### 2.10 Confocal microscopy

Imaging analyses with a confocal laser scanning microscope (LSM710NLO; Carl Zeiss, Jena, Germany) were performed as described previously (Komatsu et al., 2021). GFP and mCherry were excited with an argon laser at 488 nm and a diode-pumped solid-state laser at 561 nm, and emitted light was captured in windows of 493 and 556 nm and between 573 and 621 nm, respectively. All fluorescence images were acquired with sequential scanning, and image processing was performed with the Carl Zeiss ZEN 2012 software.

## 3 Results

### 3.1 Identification of host factors that interact with the MET domain

**Fig. 1:**
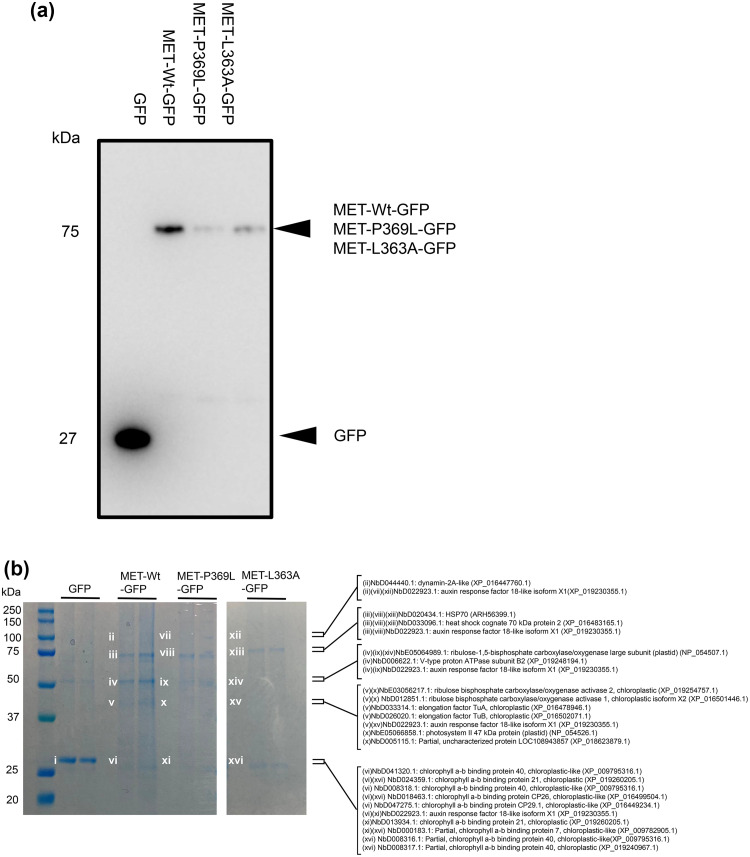
Identification of host proteins co-precipitated with the MET domain of PlAMV replicase. (a) Detection of green fluorescent protein (GFP) and GFP-fused MET domains after immunoprecipitation by Western blotting with an anti-GFP antibody. GFP, MET-Wt-GFP, MET-P369L-GFP, and MET-L363A-GFP were expressed in *Nicotiana benthamiana* and co-immunoprecipitation with GFP-trap Agarose beads was performed 24 h after agroinfiltration. Arrowheads represent expected band sizes. (b) Coomassie Brilliant Blue staining of SDS-PAGE gel loaded with samples after co-immunoprecipitation. Protein samples indicated by Roman number were excised, digested in the gel by trypsin, and analyzed by LC-MS/MS. Identified proteins with a protein score of 100 or more are shown on the right side of the image. The numbers beginning with Nb are the *N. benthamiana* database accession numbers, and the numbers in parentheses are the BLAST accession numbers for the respective proteins. Molecular weight markers are shown on the left side of the gel.

The MET domain of PlAMV replicase has a membrane-associated amphipathic helix containing amino acids that play a critical role in viral replication (Komatsu et al., 2021). We therefore used the MET domain to identify host factors involved in viral replication. We used three GFP-fused MET domain constructs: wild-type MET (MET-Wt-GFP), MET-P369L, which retains the ability to form aggregates but loses viral replication ability (MET-P369L-GFP), and MET-L363A, which lacks aggregation ability and loses viral replication ability (MET-L363A-GFP). These three types of the MET domain, as well as free GFP as a control, were transiently expressed in *N. benthamiana*, and protein extracts from infiltrated leaves were subjected to co-immunoprecipitation using GFP-trap agarose beads. Western blot analysis using an anti-GFP antibody confirmed that GFP fusion proteins were successfully recovered (Fig. 1a).

Separation of the immunoprecipitated proteins by SDS-PAGE showed that the band patterns of MET-Wt-GFP and its mutants were different from that of the free GFP control (Fig. 1b). The visible bands in the lanes of MET-Wt-GFP and those corresponding to the same molecular weight positions in the lanes of MET-P369L-GFP and MET-L363A-GFP were excised, trypsin digested, and subjected to LC-MS/MS analysis, resulting in the identification of multiple host proteins (Fig. 1b, Supplementary Table 3). HSP70 (ARH56399.1) (bands iii, viii, and xiii), which is involved in the replication of several plant viruses, including potexviruses (Wang et al., 2009; Yang et al., 2022), was co-precipitated with MET-Wt-GFP and its mutants. In contrast, V-type proton ATPase subunit B2 (XP_019248194.1) (band iv) and dynamin-2A-like (XP_016447760.1) (band ii) were co-precipitated only with MET-Wt-GFP, but not with its mutants. To investigate the mechanisms underlying MET-mediated VRC formation, we focused on the dynamin-2A-like protein, which is a membrane-localized protein similar to the MET domain and functions in membrane remodeling and trafficking. Peptide fragments detected by LC-MS/MS analysis were mapped to the entire dynamin-2A-like sequence in the BLAST database (XP_016447760.1), indicating that the whole dynamin was co-precipitated (Supplementary Fig. 1).

**Fig. 2:**
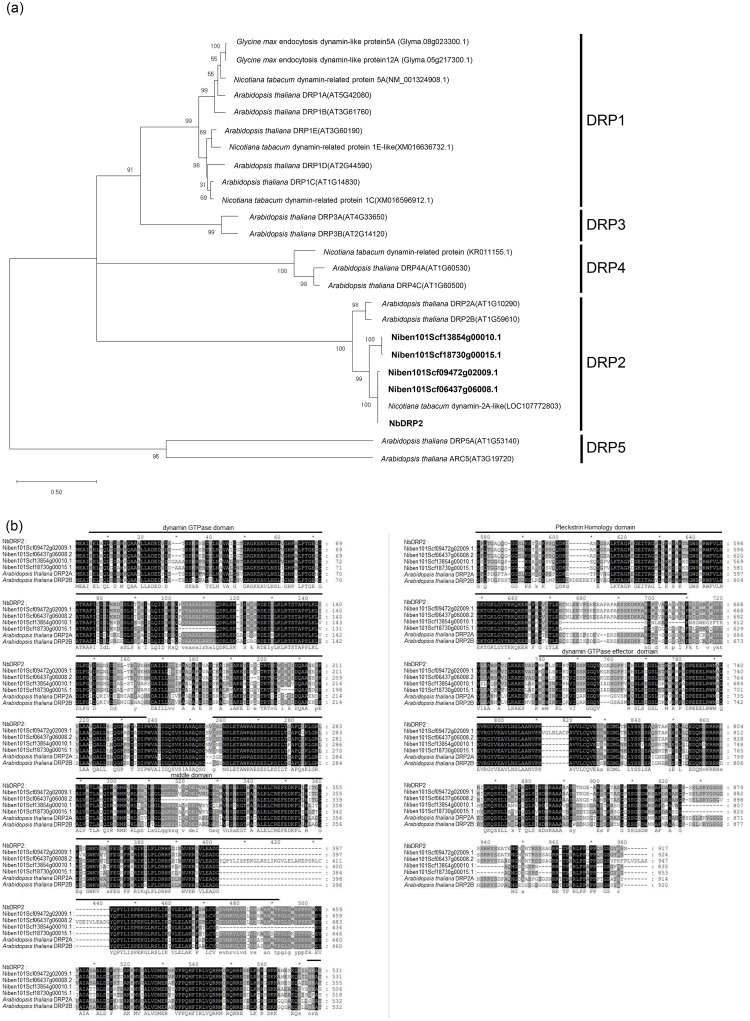
Phylogenetic analysis of plant dynamins. (a) The phylogenetic tree based on the amino acid sequences of the full-length ORF of *NbDRP2* and its homologs in *Nicotiana benthamiana* (highlighted in bold) and representative dynamin proteins. The phylogenetic tree was constructed by the maximum likelihood method using the LG+G+I model with a bootstrap replication value of 1000. Bootstrap values are shown at the nodes. The numbers in parentheses after the gene name indicate the accession numbers of the proteins. The labels on the right side of the tree indicate the five subgroups of dynamin-related proteins of *Arabidopsis thaliana* described in Hong et al. (2003). (b) The amino acid sequences of the full-length open reading frame (ORF) of NbDRP2 and its homologs, AtDRP2A and AtDRP2B, were aligned by MUSCLE. Identical and conserved amino acid residues are shaded black and dark gray, respectively. Black lines above the alignment represent characteristic domains conserved among dynamin-related proteins.

The cDNA encoding the dynamin-2A-like protein identified by mass spectrometry was amplified and cloned from *N. benthamiana* total RNA by RT-PCR. Hereinafter, we refer to this cDNA and its gene as *NbDRP2*. The full-length ORF of *NbDRP2* shared 77% and 76% amino acid identities with the *A. thaliana* dynamin-related proteins AtDRP2A and AtDRP2B, respectively, which have been reported to play a role in TuMV replication (Wu et al., 2018). BLAST analysis using the *N. benthamiana* database revealed the presence of four *NbDRP2* homologs (Niben101Scf09472g02009.1, Niben101Scf06437g06008.1, Niben101Scf18730g00015.1, and Niben101Scf13854g00010.1). Based on the ORF sequence identity to *NbDRP2*, the four homologs were divided into two groups; the high identical group includes Niben101Scf09472g02009.1 (98.3% identity) and Niben101Scf06437g06008.1 (98.9% identity), while the low identical group includes Niben101Scf18730g00015.1 (76.3% identity) and Niben101Scf13854g00010.1 (79.0% identity). Phylogenetic analysis based on the full-length amino acid sequences of the representative dynamin proteins from various plants revealed that NbDRP2 and its homologs belong to the DRP2 subfamily (Fig. 2a) (Wu et al., 2018; Fujimoto and Tsutsumi, 2014). Alignment and motif search of *N. benthamiana* dynamin proteins in this subfamily revealed that all NbDRP2 homologs possess the dynamin GTPase domain, the middle domain, the dynamin GTPase-effector domain, and the PH domain (Fig. 2b). We also examined the expression of two homologous genes of *NbDRP2*, Niben101Scf18730g00015.1 and Niben101Scf13854g00010.1, by RT-qPCR and found that these genes were not detectable in leaves (data not shown). Because we could not separately detect the expression of Niben101Scf09472g02009.1 and Niben101Scf06437g06008.1 due to their high nucleotide identity, we decided to perform subsequent experiments by referring these two genes as NbDRP2, which encode the MET domain-interacting factor.

### 3.2 MET domain interacts with NbDRP2

**Fig. 3:**
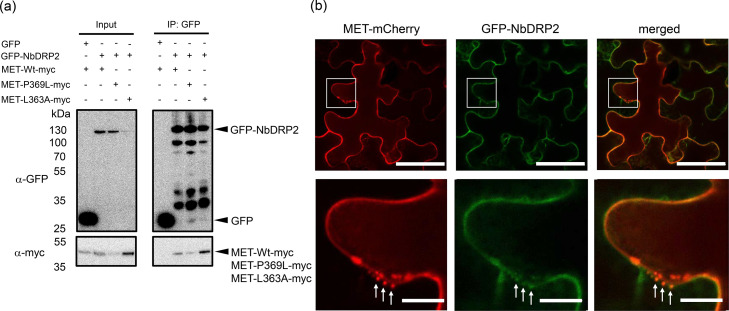
MET domain of PlAMV replicase interacts with NbDRP2. (a) Green fluorescent protein (GFP)-fused NbDRP2 (GFP-NbDRP2), myc-fused MET domain (MET-Wt-myc, MET-P369L-myc, MET-L363A-myc) and p19 silencing suppressor were co-expressed in *Nicotiana benthamiana* leaves. Total proteins were extracted at 3 days after infiltration for co-immunoprecipitation using GFP-trap agarose beads. Total protein extracts (Input) and immunoprecipitates (IP: GFP) were analyzed by Western blotting using an anti-GFP antibody and an anti-myc antibody. Arrowheads represent expected band sizes. Molecular weight is shown on the left side of the image. (b) MET-Wt-mCherry, GFP-NbDRP2, and p19 silencing suppressor was co-expressed in *Nicotiana benthamiana* leaves by agroinfiltration. Epidermal cells were observed with a confocal laser microscope at 3 days after infiltration. Lower panels show the magnified areas encompassed by squares in upper panels. Arrows indicate the positions of the spherical bodies formed by MET-mCherry. Bars in the upper and lower panels indicate 50 and 10 µm, respectively.

In co-immunoprecipitation and LC-MS/MS (IP-MS), NbDRP2 was co-precipitated only with wild-type MET domain, but not mutant MET domains. One possible reason for this is that the mutant MET domains may lack the ability to interact with NbDRP2 or that the proteins were not expressed at sufficient levels to detect the interaction. To test these possibilities, we confirmed the interaction of wild-type and mutant MET domains with NbDRP2 by co-expression with agroinfiltration and co-immunoprecipitation. The wild-type and mutant MET domains fused with the myc tag at the C-terminus (MET-Wt-myc, MET-P369L-myc, and MET-L363A-myc), NbDRP2 fused with GFP at the N-terminus (GFP-NbDRP2), and the p19 silencing suppressor were transiently coexpressed in *N. benthamiana* leaves by agroinfiltration. Three days later, protein extraction and co-immunoprecipitation with GFP-trap agarose beads were performed. Western blotting with anti-myc antibodies showed that the wild-type and both mutant MET domains co-immunoprecipitated with GFP-NbDRP2 (Fig. 3a). This result indicates that not only the wild-type MET domain, but also the MET-P369L and MET-L363A mutants interact with NbDRP2. Therefore, we decided to use only the wild-type MET domain in subsequent experiments.

Furthermore, we observed the subcellular localization of the MET domain and NbDRP2. MET domain fused to mCherry at the C-terminal end (MET-mCherry), GFP-NbDRP2 and p19 silencing suppressor were co-expressed in *N. benthamiana* leaves by agroinfiltration. Localization of the fluorescence was observed with confocal microscopy at 3 dpi. MET-mCherry localized to the cell periphery and formed small spherules, as reported previously (Komatsu et al., 2021). GFP-NbDRP2 colocalized with MET-mCherry at the cell periphery as well as the small spherules (Fig. 3b).

### 3.3 PlAMV infection induces the expression of NbDRP2

**Fig. 4:**
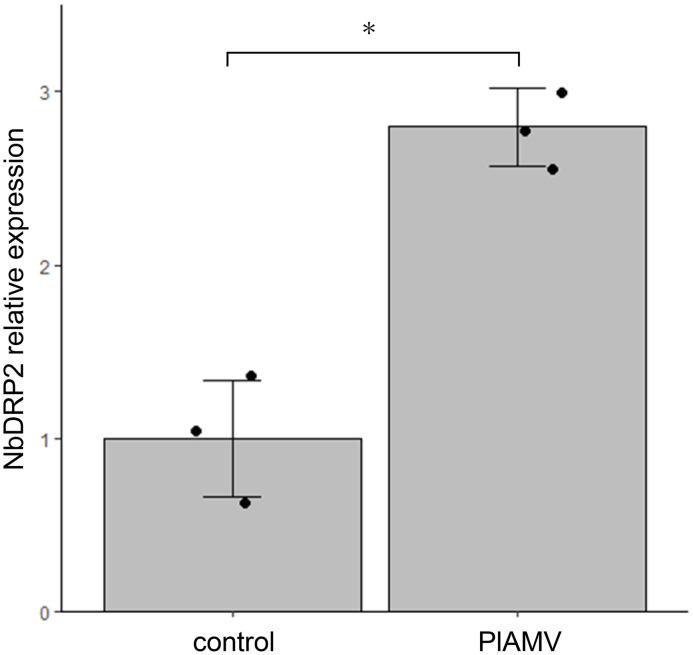
PlAMV infection induces the expression of *NbDRP2* gene. Wild-type PlAMV was inoculated into *Nicotiana benthamiana* by agroinfiltration. pCAMBIA1301 (control vector carrying the *gus* gene) was used as a control. Total RNA was extracted at 3 days after infiltration and the relative expression level of the *NbDRP2* gene was quantified by RT-qPCR using *NbPP2A* as the reference gene. The mean value of the *NbDRP2* expression level in the control is set to 1. The graph represents the mean values and the error bars indicate the standard deviation of values for three biological replicates. *n* = 3, *p < 0.05, Student's *t*-tests.

To investigate whether PlAMV infection affects *NbDRP2* expression, we examined the expression level of the endogenous *NbDRP2* gene in plants infected with PlAMV. A primer set used in this experiment was designed based on Niben101Scf09472g02009.1, and it would also amplify Niben101Scf06437g06008.1 due to its high identity. Wild-type Li1 isolate of PlAMV was inoculated on *N. benthamiana* leaves by agroinfiltration and total RNA was extracted at 3 dpi. The vector carrying the *gus* gene (pCAMBIA 1301) was used as a control. RT-qPCR results showed that the expression of *NbDRP2* was significantly upregulated in virus-inoculated leaves compared to control leaves (Fig. 4).

### 3.4 PlAMV co-opts NbDRP2, essential for plant growth, for viral replication

**Fig. 5:**
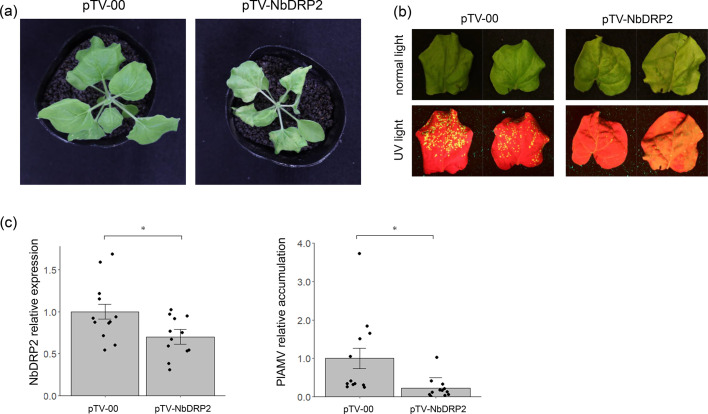
Knockdown of *NbDRP2* gene reduces PlAMV accumulation. (a) Representative images of *Nicotiana benthamiana* plants inoculated with the TRV vector pTV-00 (empty vector) or pTV-NbDRP2. Images were taken at 10 days after TRV inoculation. (b) Representative images of PlAMV-GFP inoculated leaves of the pTV-00- or pTV-NbDRP2-infected plants at 3 dpi. Two representative leaves are shown. (c) Quantification of *NbDRP2* gene expression and virus accumulation in PlAMV-GFP inoculated leaves by RT-qPCR. The reference gene is *NbPP2A*. The mean value of *NbDRP2* gene expression and virus accumulation in the pTV-00 (control) group was set as 1. Data represent mean values ± standard error of the mean calculated from three independent experiments, each with four biological replicates by using a mixed effects model (*p < 0.05).

To analyze the function of NbDRP2 in PlAMV infection, we attempted to knockdown *NbDRP2* gene in *N. benthamiana* by virus-induced gene silencing (VIGS) using a tobacco rattle virus (TRV) vector. We designed the VIGS target sequence that can knockdown *NbDRP2* gene using the nucleotide sequence of Niben101Scf09472g02009.1 as a search query for the pssRNAit program (Supplementary Fig. 2). At 10 days after inoculation of the TRV carrying the *NbDRP2* fragment (pTV-NbDRP2) or the TRV vector (pTV-00), PlAMV-GFP was mechanically inoculated onto upper uninoculated leaves. At this time point, pTV-NbDRP2-infected plants exhibited growth inhibition with leaf malformation (Fig. 5a). Fluorescence observation and RT-qPCR analysis were performed at 3 dpi when the knockdown of *NbDRP2* gene in the PlAMV-GFP inoculated leaves was confirmed (Fig. 5c, left). GFP intensity in the inoculated leaves was impaired in *NbDRP2* knockdown plants compared to control plants (Fig. 5b). Consistent with this, the accumulation level of PlAMV-GFP was decreased significantly in *NbDRP2*-knockdown plants compared to the control plants (Fig. 5c, right).

In the VIGS experiment, the reduction in plant growth may have affected virus accumulation. Therefore, we performed the same experiments with ToMV (TocJ/GFP) and CMV, which have not been reported to use dynamin for virus replication. RT-qPCR showed that the accumulation level of both TocJ/GFP and CMV tended to be lower in NbDRP2-VIGS plants (Supplementary Fig. 3, 4). These results suggest the possibility that NbDRP2 is also involved in the replication of these viruses, but do not rule out the possibility that reduced plant growth affects virus replication. Therefore, we performed the transient knockdown of the *NbDRP2* gene, a method that has less effect on plant growth, by expressing a hairpin construct that contains the same target sequence as VIGS into *N. benthamiana* leaves by agroinfiltration (Brendolise et al., 2017). Two days after agroinfiltration, PlAMV virions of Li1 isolate without GFP were mechanically inoculated on agroinfiltrated leaves. No phenotypes such as necrosis were observed in agroinfiltrated regions with hairpin constructs, and plant growth was normal and did not differ from control plants (Supplementary Fig. 5a, b). Although not significantly different, the expression level of *NbDRP2* in leaves agroinfiltrated with the NbDRP2-hairpin construct was lower than in controls (Supplementary Fig. 5c, left), and PlAMV accumulation also tended to decrease (Supplementary Fig. 5c, right). These results indicate that PlAMV accumulation is suppressed in NbDRP2 knockdown plants regardless of plant growth conditions.

Taking advantage of the ability of PlAMV-GFP to effectively infect *A. thaliana*, we further investigated whether the *A. thaliana* DRP2 subfamily proteins AtDRP2A and AtDRP2B, which are NbDRP2 homologs, play a proviral role in PlAMV infection. Wild-type *A. thaliana* (Col-0) and the *atdrp2a* and *atdrp2b* mutants were inoculated with PlAMV-GFP. Fluorescence observation of the inoculated leaves at 2 dpi showed that the number of GFP spots on *atdrp2a* and *atdrp2b* was comparable to that of Col-0 (Supplementary Fig. 6). Although we could not test the infectivity of PlAMV-GFP in the *atdrp2a* and *atdrp2b* double mutant due to its lethality (Backues et al., 2010), these results suggest that the functions of AtDRP2A and AtDRP2B in PlAMV infection are redundant or that AtDRP2A and AtDRP2B are not involved in PlAMV infection in *A. thaliana*.

### 3.5 NbDRP2 promotes PlAMV accumulation at the single-cell level

**Fig. 6:**
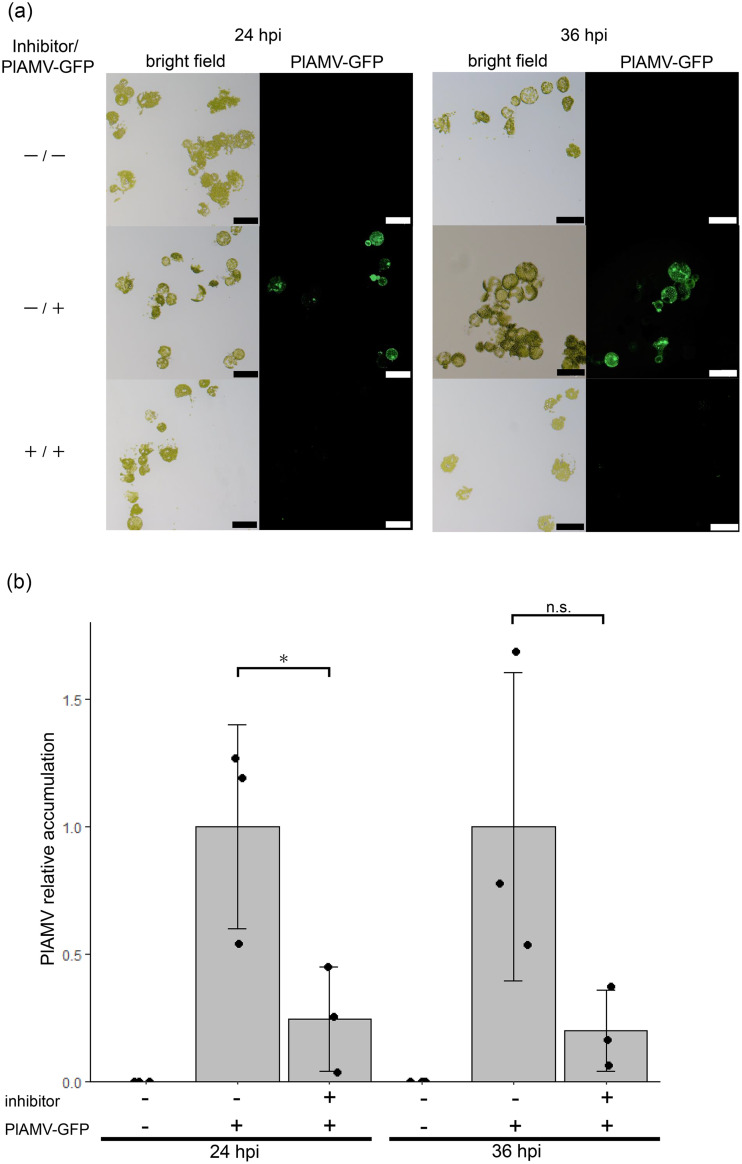
Chemical inhibition of dynamin function suppresses virus accumulation in protoplasts. (a) Dynasore, an inhibitor of the GTPase activity of dynamin, or DMSO as a control, was added to the culture medium of protoplasts prepared from *Nicotiana benthamiana* leaves at a final concentration of 100 µM before and after PlAMV-GFP or water inoculation, and fluorescence observation was performed at 24 and 36 h after inoculation. The scale bar represents 100 µm. (b) RNA was extracted from protoplasts after 24 hpi and 36 hpi, respectively, and virus accumulation was quantified by absolute RT-qPCR. The mean value of virus accumulation in the inhibitor-untreated and virus-inoculated (-/+) group was set to 1. The graph represents the mean values with error bars indicating the standard deviation of the values for three biological replicates. *n* = 3, * *p*<0.05, n.s.: not significant by the Student's *t*-test.

To analyze a role of NbDRP2 in PlAMV replication at the single-cell level, we examined virus accumulation in protoplasts treated with Dynasore, a chemical inhibitor of the GTPase activity of dynamin. *N. benthamiana* protoplasts were treated with Dynasore or DMSO as a control at a final concentration of 100 µM in the culture medium before and after the inoculation with the *in vitro* transcribed RNA of PlAMV-GFP. At 24 and 36 h after viral inoculation, Dynasore treatment had no effect on protoplast viability (Supplementary Fig. 7) but resulted in a remarkable decrease in GFP fluorescence in the inhibitor-treated protoplasts, indicating suppressed viral replication (Fig. 6a). Furthermore, RT-qPCR confirmed that viral RNA accumulation levels were lower in the inhibitor-treated protoplasts than in the inhibitor-untreated protoplasts (Fig. 6b). These results indicated that inhibition of dynamin GTPase activity impaired viral replication.

## 4 Discussion

In this study, we identified NbDRP2 as a host factor that interacts with the MET domain of PlAMV replicase in *N. benthamiana* by co-immunoprecipitation and LC-MS/MS (IP-MS) analysis. Our previous study has shown that mutations into the membrane-associated helix in the MET domain, P369L and L363A, were deleterious for PlAMV replication (Komatsu et al., 2021). However, co-immunoprecipitation analysis revealed that MET-P369L and MET-L363A also interact with NbDRP2, indicating that these mutations do not affect the interaction between the MET domain and NbDRP2. Therefore, it is possible that interactions with other host factors identified by IP-MS in this study or structural changes due to the amino acid mutations may be involved in the loss of viral replication by P369L and L363A.

In addition to NbDRP2, IP-MS detected other host factors reported to be involved in viral replication, including heat shock protein 70 (Hsp70) and V-type proton ATPase subunit B2. The molecular chaperone Hsp70 is involved in the subcellular localization of the TBSV replication proteins p33 and p92^pol^ and their insertion into the intracellular membranes (Wang et al., 2009). The interaction of the RCNMV replication proteins p27 and Hsp70 on the ER membrane is also required for VRC formation (Mine et al., 2012). It has also been shown that infection with tobacco mosaic virus, potato virus X, cucumber mosaic virus and watermelon mosaic virus upregulate *Hsp70* gene expression to facilitate virus accumulation and movement (Chen et al., 2008). Indeed, the replicase of bamboo mosaic virus (BaMV), a member of the same genus *Potexvirus* as PlAMV, interacts with Hsp70, which is involved in viral replication (Huang et al., 2017). Additionally, CNV has been found to use a homolog of Hsp70: Hsc70 for viral infection (Alam and Rochon, 2017). Taken together, the identification of Hsp70 as an interactor of the PlAMV replicase is consistent with these previous reports. The V-type proton ATPase, a vacuolar ATPase, induces vacuolar membrane acidification and autophagy through the interaction between catalytic subunit B2 (VHA-B2) and subunit E (VHA-E). Barley stripe mosaic virus (BSMV) replication protein γa binds to VHA-B2 via its Arg-569 residue to inhibit the interaction between VHA-B2 and VHA-E, suppressing vacuolar acidification and promoting virus infection (Yang et al., 2022). Thus, not only NbDRP2 but also several host factors interacting with the MET domain of the PlAMV replicase may promote viral replication by inducing VRC formation and suppressing antiviral responses, although further investigation is required to confirm this hypothesis.

Identification of several host factors interacting with the MET domain suggests that the MET domain may be important for the recruitment of host factors to the VRC, in addition to its role in membrane-association. This assumption is consistent with recent findings on replicase “crown” structure in infected cells. Similar to the MET domain of potexviruses, non-structural protein 1 (nsP1) of chikungunya virus (CHKIV), which belongs to the alphavirus-like superfamily that includes the genus *Potexvirus*, has N7-guanine-methyltransferase and guanylyltransferase activities and is a viral RNA capping protein. nsP1 is responsible for membrane association and has recently been found to form crown structures at the cellular membrane and to play a role as the basis for the VRC formation (Zhang et al., 2021). Therefore, viral proteins with methyltransferase and capping activities may generally play a role in the formation of the basic structure that is important for the interaction with host factors required for VRC formation.

PlAMV accumulation was decreased by knockdown of *NbDRP2* gene by VIGS. Furthermore, PlAMV infection induced *NbDRP2* gene expression. These results suggest that NbDRP2 is a proviral factor for PlAMV replication. Phylogenetic analysis revealed that NbDRP2 and its homologs have high identity with AtDRP2A and AtDRP2B of *A. thaliana*, each of which is involved in TuMV replication (Wu et al., 2018). It is therefore possible that the DRP2 subfamily proteins are widely used for replication of (+)ssRNA viruses. Also, our virus inoculation tests on *A. thaliana atdrp2a* and *atdrp2b* single mutants showed that the number of fluorescent spots of PlAMV-GFP was comparable between the wild-type and these single mutants. This result differs from the previous study showing that TuMV accumulation was reduced in *atdrp2a* or *atdrp2b* single mutants (Wu et al., 2018). This suggests that both AtDRP2A and AtDRP2B are required for TuMV replication, whereas only one of them is sufficient for PlAMV replication and their proviral functions are redundant. Further analysis is required to reveal whether and why the requirement for DRP2s in viral replication differs between viruses. However, we cannot exclude the possibility that AtDRP2A and AtDRP2B are not involved in PlAMV infection. If this is the case, it is in sharp contrast to our finding that NbDRP2 is an important host factor for PlAMV replication in *N. benthamiana*, suggesting that the host factors used for PlAMV replication may vary between plant species. However, based on the fact that NbDRP2 was classified in the same group as AtDRP2A and AtDRP2B in our phylogenetic analysis, we would prefer to conclude that they are involved in PlAMV replication, and their functions may be redundant. Further studies are needed to clarify which explanation is more likely, including analysis of the interactions between the virus replicase and host factors in there two plants.

Furthermore, the inhibition of plant growth by VIGS of *NbDRP2* gene indicates its importance in plant development, which is consistent with the lethality of the *atdrp2a* and *atdrp2b* double mutant of *A. thaliana* (Backues et al., 2010). However, the result of the transient knockdown of *NbDRP2* gene experiment and the fact that no abnormality was observed in the protoplasts treated with dynamin inhibitor, as well as there was no difference in the viability of the cells from the control, indicate that the reduced PlAMV accumulation in NbDRP2-VIGS plants was not due to the weakened plant growth. Altogether, the fact that PlAMV accumulation was significantly reduced in NbDRP2-VIGS plants suggests the possibility that PlAMV establishes virus replication by exploiting the intrinsic function of DRP2 subfamily proteins. Since there are some functional differences between AtDRP2A and AtDRP2B in *A. thaliana* (Smith et al., 2014), it is also of interest to investigate the correspondence between these homologs in *N. benthamiana* and their function in virus replication.

In this study, treatment of protoplasts with dynamin inhibitor effectively reduced PlAMV accumulation by inhibiting the GTPase activity of DRP1 and DRP2, both of which have GTPase active domains. In a previous study, AtDRP1 was reported to play a proviral role in TuMV replication (Wu et al., 2020). In *A. thaliana*, DRP1 and DRP2 localize to the plasma membrane and cell plate, with DRP2 additionally localizing to the Golgi/trans-Golgi network (TGN)/endosomes involved in the post-Golgi pathway (Fujimoto and Tsutsumi, 2014). Both proteins are involved in mitosis and act in endocytosis in a coordinated manner (Fujimoto and Tsutsumi, 2014). It is therefore possible that endocytosis, in which DRP1 and DRP2 act coordinately may be involved in virus replication. Moreover, it is unclear whether DRP1 and DRP2 have separate roles in virus replication or not. The relevance of the subcellular localization and functions of DRP1 and DRP2 in viral replication requires further investigation.

It also remains to be elucidated where and how NbDRP2 is involved in viral replication. Plant cells contain insoluble membrane microdomains called lipid rafts, which consist of sphingolipids, cholesterol, and membrane-localized proteins and are involved in intracellular signaling, protein trafficking, and pathogen infection (Martinière and Zelazny, 2021). A previous study reported that lipid rafts in tobacco BY2 cells contain dynamin-like proteins (Mongrand et al., 2004). In addition, plant viruses recruit host factors to the replication site to form membrane microdomains, called membrane contact sites (MCS), to promote VRC formation (Barajas et al., 2014). The MET domain forms small spherules in close association with the ER membrane (Komatsu et al., 2021), and ER is a part of the vesicular trafficking pathway in which DRP2 is involved. Confocal microscopy observation also showed that NbDRP2 colocalizes with the spherules of the MET domain. Therefore, it is tempting to speculate that these small spherules are associated with MCSs and that NbDRP2 is a component of membrane microdomains and serves as a scaffold to create a membrane microenvironment suitable for VRC formation in PlAMV replication.

In this study, we identified NbDRP2, a proviral host factor that interacts with the MET domain of the PlAMV replicase. Based on the previous reports that the membrane-associated MET domain, which is conserved in many alphaviruses, is important for the interaction with host factors, it is possible that the interaction between the MET domain in viral replicase and DRPs is common in at least related (+)ssRNA viruses ("Branch 3″ of RdRp, Wolf et al., 2018). Future studies are needed to determine whether DRP2 is involved in replication of other virus species. Also, investigation of other DRP subfamilies that share a common domain structure with the DRP2 subfamily may lead to a better understanding of the mechanism of viral replication and VRC formation.

## 5 Conclusions

Dynamin-related protein 2 in *N. benthamiana* (NbDRP2) is a host factor that interacts with the MET domain of PlAMV replicase. PlAMV infection induced the expression of the *NbDRP2* gene, and the PlAMV accumulation was reduced in NbDRP2-knock down plants and dynamin inhibitor treated protoplasts. Altogether, it is suggested that NbDRP2 has a proviral role in PlAMV replication.

## Data availability

Data will be made available on request.

## Funding

This work was partially supported by a grant-in-aid for scientific research (C) from the Japan Society for the Promotion of Sciences (JSPS) (number 19K06048) to K. Komatsu, a grant-in-aid for 10.13039/501100001691JSPS fellow (number 20F20392) to I. Hamim, and the Special Research Fund of the Institute of Global Innovation Research at Tokyo University of Agriculture and Technology (GIR-TUAT) to K. Komatsu and N. Sasaki.

## CRediT authorship contribution statement

**Haruka Shinji:** Methodology, Investigation, Software, Validation, Visualization, Data curation, Formal analysis, Writing – original draft, Writing – review & editing. **Nobumitsu Sasaki:** Methodology, Investigation, Resources, Validation, Writing – review & editing. **Islam Hamim:** Investigation, Writing – review & editing. **Yoshiyuki Itoh:** Investigation, Writing – review & editing. **Kazuo Taku:** Investigation. **Yuho Hayashi:** Methodology, Investigation. **Nami Minato:** Writing – review & editing. **Hiromitsu Moriyama:** Methodology, Writing – review & editing. **Tsutomu Arie:** Supervision, Writing – review & editing. **Ken Komatsu:** Conceptualization, Methodology, Resources, Supervision, Validation, Funding acquisition, Writing – original draft, Writing – review & editing.

## Declaration of Competing Interest

The authors declare that they have no known competing financial interests or personal relationships that could have appeared to influence the work reported in this paper.

## Appendix Supplementary materials

Image, application 1

Image, application 2

Image, application 3

Supplementary materials Supplementary material associated with this article can be found in the online version. Image, application 4
