# Supplementary material for: Dynamin-related protein 2 interacts with the membrane-associated methyltransferase domain of plantago asiatica mosaic virus replicase and promotes viral replication
Source: Virus Res. 2023 May 14;331:199128. doi: 10.1016/j.virusres.2023.199128 (PMC10345747; doi:10.1016/j.virusres.2023.199128)
Supplement: Supplementary file 1 [file mmc1.pdf]

NbD044440.1: dynamin-2A-like (XP_016447760.1)

MEAIEELAQLSDSMKQAAALLADEDVDETSSKRSSTFLNVVAIGGTGAGKSAVLNSLVGHPALPTGEGGATRAPICIDLKRDSSLSTKSIVLQIDSKSQPVSAS
ALRHSLQDRLSKISSKSRDEIYLKLRTSTAPPLKLIDLPGVDKGNLDDSLTEYVEHNDAILLVVISAAQAPEVASCKAIRIAKEYDSECTRTVGVISKIDQAASEP
KVLAAVQALLSGQGPRSTADIPWVALIGQSVSIASAQSGSVGSDNSLETAWRAESESLKSILTGAPQSKLGRLALVETLAHQIRNRMKVRLPNLLSGLQGKS
QVVQDELVRLGEQMVNSAEGTKALALELCREFEDKFLLHITGGEGDGWKVVASFEGNFPNRIKQLPLDRHFDINNVKRIVLEADGYQPYLISPEKGLRSLIKG
VLELAKEPSRLCVDEVHRVLVDLVSSAANATPGLGRYPPFKREVVAIASAALDGFKTDAKKMVVALVDMERAFVPPQHFIRLVQRRMDRQRREDELKNRGS
KKAHESEQSILNRATSPQTGAQQGGGSLKSMKEKPSQQDKDASEGSALKTAGPEGEITAGFLLKRSAKTNGWSKRWFVLNEKTGKLGYTKKQEERHFRG
VITLEECNLEEVPDEEEAPAPAKSSKDKKANGPDVAKAPNLVFKITSRVPYKTVLKAHSAVILKAESVADKMEWLSKLRTVISSKGGQVKGESGPPIRHSLSD
GSLETMTRRPVDPEEELRWMAQEVRGYVEAVLNSLAANVPKAVVLCQVEKAKEDMLTKLYSSISAQSTAKIEELLQEDQNVKRRRERIQKQSSLLSKLTRQL
SIHDNRAAAAASYANGEAESSPTASGPSSGDDWRSAFDAAANGPSSLSRYGSGGSSRRYSEAAENGNANTRSSSAGRRTPNRLPPGPPQSGSGYRS

Supplementary Figure 1: Mapping of the peptides detected by LC-MS/MS analysis to the amino acid sequences of the dynamin-2A-like protein (XP_016447760.1).

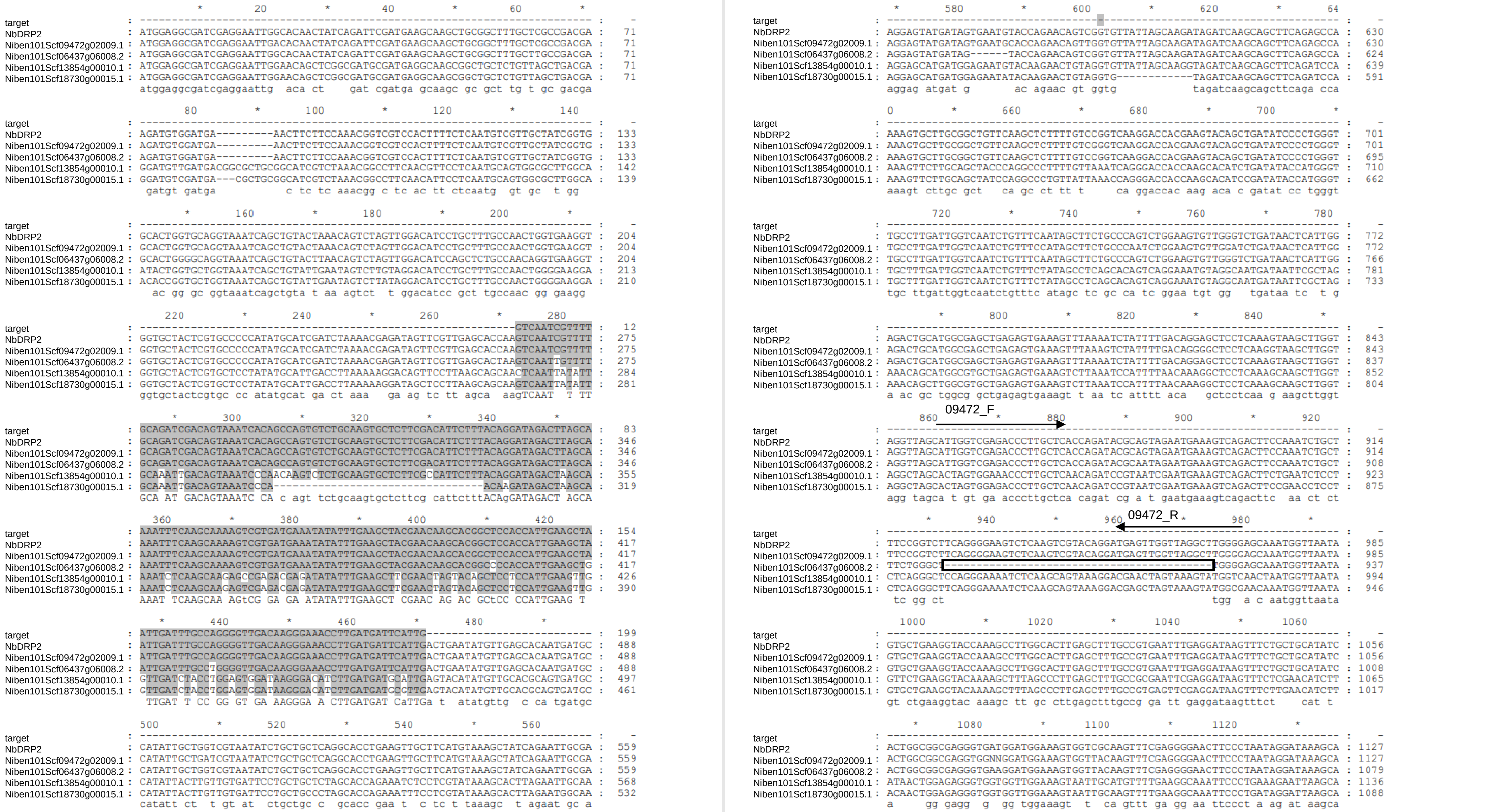

09472_F
09472_R
target
NbDRP2
Niben101Scf09472g02009.1
Niben101Scf06437g06008.2
Niben101Scf13854g00010.1
Niben101Scf18730g00015.1

```
                           1140         *      1160         *      1180         *      1200
target                   : ----------------------------------------------------------------------- :    -
NbDRP2                   : GCTCCCCTTAGATAGACATTTTGACATAAACAATGTCAAGCG----------------------------- : 1169
Niben101Scf09472g02009.1 : GCTCCCCTTAGATAGACATTTTGACATAAACAATGTCAAGCG----------------------------- : 1169
Niben101Scf06437g06008.2 : GCTCCCCTTAGATAGACATTTTGACATAAACAATGTCAAGCGGATTGTGTTAGAAGCCGATGGTTATCAGC : 1150
Niben101Scf13854g00010.1 : GCTTCCTTTGGATAGACACTTTGAGTTAAAAAATGTGAAGAG----------------------------- : 1178
Niben101Scf18730g00015.1 : GCTTCCTTTGGATAGACACTTTGAGTTAAAAAATGTGAAGAG----------------------------- : 1130
                           gct cc tt gatagaca tttga  taaa aatgt aag g

                             *      1220         *      1240         *      1260         *      12
target                   : ----------------------------------------------------------------------- :    -
NbDRP2                   : ----------------------------------------------------------------------- :    -
Niben101Scf09472g02009.1 : ----------------------------------------------------------------------- :    -
Niben101Scf06437g06008.2 : CTTACCTGATTTCTCCCGAGAAAGGGTTGAGGTCTTTGATAAAAGGTGTCCTGGAGCTTGCAAAAGAACCT : 1221
Niben101Scf13854g00010.1 : ----------------------------------------------------------------------- :    -
Niben101Scf18730g00015.1 : ----------------------------------------------------------------------- :    -

                           80         *      1300         *      1320         *      1340
target                   : ----------------------------------------------------------------------- :    -
NbDRP2                   : --------------------GATTGTGTTAGAAGCCGATGGTTATCAGCCTTACCTGATTTCTCCCGAGAA : 1220
Niben101Scf09472g02009.1 : --------------------GATTGTGTTAGAAGCCGATGGTTATCAGCCTTACCTGATTTCTCCCGAGAA : 1220
Niben101Scf06437g06008.2 : TCACGTCTTTGTGTTGATGAGATTGTGTTAGAAGCCGATGGTTATCAGCCTTACCTGATTTCTCCCGAGAA : 1292
Niben101Scf13854g00010.1 : --------------------GGTTGTGCTAGAAGCAGATGGCTATCAACCTTACCTTATTTCTCCTGAAAA : 1229
Niben101Scf18730g00015.1 : --------------------GGTTGTGCTAGAAGCAGATGGCTATCAACCTTACCTTATTTCTCCTGAAAA : 1181
                                               g ttgtg tagaagc gatgg tatca ccttacct atttctcc ga aa

                           *      1360         *      1380         *      1400         *      1420
target                   : ----------------------------------------------------------------------- :    -
NbDRP2                   : AGGGTTGAGGTCTTTGATAAAAGGTGTCCTGGAGCTTGCAAAAGAACCTTCACGTCTTTGTGTTGATGAGG : 1291
Niben101Scf09472g02009.1 : AGGGTTGAGGTCTTTGATAAAAGGTGTCCTGGAGCTTGCAAAAGAACCTTCACGTCTTTGTGTTGATGAGG : 1291
Niben101Scf06437g06008.2 : AGGGTTGAGGTCTTTGATAAAAGGTGTCCTGGAGCTTGCAAAAGAACCTTCACGTCTTTGTGTTGATGAGG : 1363
Niben101Scf13854g00010.1 : AGGGTTAAGGTCTTTAATAAAGAGTGTATTGGAGCTGGCTAAAGAACCTTCAACACTTTGTGTCGA----- : 1295
Niben101Scf18730g00015.1 : AGGGTTAAGGTCTTTAATAAAGGGTGTATTAGAGCTGGCTAAAGAACCTTCAACACTTTGTGTCGAAGAGG : 1252
                           agggtt aggtcttt ataaa  gtgt  t gagct gc aaagaaccttca   ctttgtgt ga

                                    *      1440         *      1460         *      1480         *
target                   : ----------------------------------------------------------------------- :    -
NbDRP2                   : TGCACCGTGTACTTGTTGATCTTGTCTCCTCTGCTGCAAATGCCACACCTGGACTTGGACGATATCCTCCT : 1362
Niben101Scf09472g02009.1 : TGCACCGTGTACTTGTTGATCTTGTCTCCTCTGCTGCAAATGCCACACCTGGACTTGGACGATATCCTCCT : 1362
Niben101Scf06437g06008.2 : TGCACCGTGTACTTGTTGATCTTGTCTCCTCTGCTGCAAATGCCACACCTGGACTTGGACGATATCCTCCT : 1434
Niben101Scf13854g00010.1 : ----------------------------------------------------------------------- :    -
Niben101Scf18730g00015.1 : TGCACCGAGTACTTGTTGATATTGTCTCAAAAGCTGCTAATTCAACACCAGGTCTTGGAAAGTATCCTCCT : 1323

                                1500         *      1520         *      1540         *      1560
target                   : ----------------------------------------------------------------------- :    -
NbDRP2                   : TTCAAGAGAGAGGTTGTAGCAATTGCTTCTGCTGCATTGGATGGGTTTAAAACTGATGCCAAGAAAATGGT : 1433
Niben101Scf09472g02009.1 : TTCAAGAGAGAGGTTGTAGCAATTGCTTCTGCTGCATTGGATGGGTTTAAAACTGATGCCAAGAAAATGGT : 1433
Niben101Scf06437g06008.2 : TTCAAGAGAGAGGTTGTAGCAATTGCTTCTGCTGCATTGGATGGGTTTAAAACTGATGCCAAGAAAATGGT : 1505
Niben101Scf13854g00010.1 : --------AGAGGTAATTGCAATTGCGACTACTGCTTTGGATAATTTTAGAACTGAAGCAAAGAATATGGT : 1358
Niben101Scf18730g00015.1 : TTTAAGCAAGAGGTAATTGCAATTGCGACTACTGCTTTGGATAGTTTTAGAACTGAAGCGAAGAATATGGT : 1394
                                   agaggt  t gcaattgc  ct ctgc ttggat   ttta aactga gc aagaa atggt

                                  *      1580         *      1600         *      1620         *
target                   : ----------------------------------------------------------------------- :    -
NbDRP2                   : AGTTGCCCTTGTCGACATGGAGCGAGTTTTTGTCCCCCCTCAACACTTTATCCGCTTGGTGCAGAGGAGAA : 1504
Niben101Scf09472g02009.1 : AGTTGCCCTTGTCGACATGGAGCGAGTTTTTGTCCCCCCTCAACACTTTATCCGCTTGGTGCAGAGGAGAA : 1504
Niben101Scf06437g06008.2 : AGTTGCCCTTGTCGACATGGAGCGAGCTTTTGTCCCTCCTCAACACTTTATCCGCTTGGTGCAGAGGAGAA : 1576
Niben101Scf13854g00010.1 : TATTGCCCTTGTTGACATGGAGCGGGTGTATGTTCCCCCACAACACTTTATCCGTTTGGTGCAAAGGCGGA : 1429
Niben101Scf18730g00015.1 : TATTGCCCTTGTTGACATGGAGCGGGTGTATGTTCCCCCACAGCACTTTATCCGTTTGGTGCAAAGGCGGA : 1465
                             ttgcccttgt gacatggagcg g  t tgt cc cc ca cactttatccg ttggtgca agg g a

                              1640         *      1660         *      1680         *      1700
target                   : ----------------------------------------------------------------------- :    -
NbDRP2                   : TGGACAGACAGAGACGAGAAGATGAGCTAAAAAATCGGGGTTCGAAGAAGGCACATGAATCAGAGCAATCA : 1575
Niben101Scf09472g02009.1 : TGGACAGACAGAGACGAGAAGATGAGCTAAAAAATCGGGGTTCGAAGAAGGCACATGAATCAGAGCAATCA : 1575
Niben101Scf06437g06008.2 : TGGACAGACAGAGACGAGAGGATGAGCTAAAGAATCGGGGTTCGAAGAAGGCACATGAATCAGAGCAAGCA : 1647
Niben101Scf13854g00010.1 : TGGATAGGCAGCGGCGAGAGGACGACCTGAAGAACCGACCCTCCAAGAAGGCAGCAGAGGCAGAGCAATCC : 1500
Niben101Scf18730g00015.1 : TGGATAGGCAGCGGCGAGAGGACGACCTGAAGACTCGACCCTCCAAGAAGGCAGCAGAGGCAGAGCAATCC : 1536
                           tgga ag cag g cgaga ga ga ct aa a  cg    tc aagaaggca   ga  cagagcaa c

                                *      1720         *      1740         *      1760         *
target                   : ----------------------------------------------------------------------- :    -
NbDRP2                   : ATATTGAATAGGGCAACTAGTCCTCAAACTGGAGCCCAGCAAGGAGGAGGAAGCTTGAAATCTACGAAAGA : 1646
Niben101Scf09472g02009.1 : ATATTGAATAGGGCAACTAGTCCTCAAACTGGAGCCCAGCAAGGAGGAGGAAGCTTGAAATCTACGAAAGA : 1646
Niben101Scf06437g06008.2 : ATATTGAATAGGGCAACTAGTCCTCAAACTGGAGCCCAGCAAGGTGGAGGAAGCTTGAAATCTATGAAAGA : 1718
Niben101Scf13854g00010.1 : GTATCGAATAGGGCATCCAGTTCCCAAACTGGAGGTGAACAAAGTGGTGGA------AAGTCGGCGAAGGA : 1565
Niben101Scf18730g00015.1 : ATATCGAATAAGGCATCCAGTTCCCAAACCGGAGGTGAACAAAGTGGTGGA------AAGTCAGCGAAGGA : 1601
                            tat gaata ggca c agt c caaac ggag   a caa g gg gga      aa tc   gaa ga

                            1780         *      1800         *      1820         *      1840
target                   : ----------------------------------------------------------------------- :    -
NbDRP2                   : AAAACCCAGCCAGCAGGACAAGGATGCATCAGAAGGCTCGGCTTTGAAAACAGCAGGGCCTGAGGGAGAAA : 1717
Niben101Scf09472g02009.1 : AAAACCCAGCCAGCAGGACAAGGATGCATCAGAAGGCTCGGCTTTGAAAACAGCAGGGCCTGAGGGAGAAA : 1717
Niben101Scf06437g06008.2 : AAAACCCAGCCAGCAGGACAAGGATGCATCAGAAGGCTCGGCTTTGAAAACAGCAGGGCCTGAGGGAGAAA : 1789
Niben101Scf13854g00010.1 : TAAATCAGTTCAGCAAGATAAGGATTCACAAGAAGGACCAGTCCTGAAGACTGCAGGACCCGATGGGGAAA : 1636
Niben101Scf18730g00015.1 : TAAATCTGTTCAGCAAGATAAGGATTCACAAGAAGGACCAGTCCTGAAGACTGCAGGACCTGATGGGGAAA : 1672
                            aaa c    cagca ga aaggat ca  agaagg  c g   tgaa ac gcagg cc ga gg gaaa

                              *      1860         *      1880         *      1900         *      1
target                   : ----------------------------------------------------------------------- :    -
NbDRP2                   : TTACAGCTGGTTTCTTATTGAAGAGAAGTGCCAAAACAAATGGGTGGAGTAAGCGATGGTTTGTTTTGAAT : 1788
Niben101Scf09472g02009.1 : TTACAGCTGGTTTCTTATTGAAGAGAAGTGCCAAAACAAATGGGTGGAGTAAGCGATGGTTTGTTTTGAAT : 1788
Niben101Scf06437g06008.2 : TTACAGCTGGTTTCTTATTGAAGAGAAGTGCCAAAACAAATGGGTGGAGTAAGCGATGGTTTGTTTTGAAT : 1860
Niben101Scf13854g00010.1 : TCACAGCAGGGTTTCTACTAAAGAAAAGTGATAAAAAAAGTGGCTGGAGCAAGCGATGGTTTGTTTTAAAT : 1707
Niben101Scf18730g00015.1 : TCACAGCAGGGTTTCTACTAAAGAAAAGTGATAAAAAAAGTGGCTGGAGCAAGCGATGGTTTGTTTTAAAT : 1743
                           t acagc gg tt  ta t aaga aagtg  aaaa aa tgg tggag aagcgatggtttgtttt aat

                           920         *      1940         *      1960         *      1980
target                   : ----------------------------------------------------------------------- :    -
NbDRP2                   : GAGAAAACCGGAAAGCTTGGATACACGAAGAAACAAGAAGAACGTCATTTTCGTGGTGTCATAACGTTGGA : 1859
Niben101Scf09472g02009.1 : GAGAAAACTGGAAAGCTTGGATACACGAAGAAACAAGAAGAACGTCATTTTCGTGGTGTCATAACATTGGA : 1859
Niben101Scf06437g06008.2 : GAGAAAACCGGAAAGCTTGGATACACGAAGAAACAAGAAGAACGCCATTTTCGTGGTGTCATAACGTTGGA : 1931
Niben101Scf13854g00010.1 : GAGAAGACTGGAAAGCTTGGATATACCAAGAAGCAAGAAGAGCGGCATTTCCATGGTGTAATTACGTTGG- : 1777
Niben101Scf18730g00015.1 : GAGAAGACTGGAAAGCTTGGGTATACAAAGAAGCAAGAAGAGCGCCATTTCCATGGTGTCATTACGTTGG- : 1813
                           gagaa ac ggaaagcttgg ta ac aagaa caagaaga cg cattt c tggtgt at ac ttgg

                            *      2000         *      2020         *      2040         *      206
target                   : ----------------------------------------------------------------------- :    -
NbDRP2                   : GGAATGTAATCTTGAAGAAGTTCCTGATGAAGAAGAAGCTCCAGCACCTGCCAAAAGTTCCAAGGACAAAA : 1930
Niben101Scf09472g02009.1 : GGAATGTAATCTTGAAGAAGTTCCTGATGAAGAAGAAGCTCCGGCACCTGCCAAAAGTTCCAAGGACAAAA : 1930
Niben101Scf06437g06008.2 : GGAATGTAATCTTGAAGAAGTTCCTGATGAAGAAGAAGCTCCAGCACCTGCCAAAAGTTCCAAGGACAAAA : 2002
Niben101Scf13854g00010.1 : ----------------------------------------------------------------------- :    -
Niben101Scf18730g00015.1 : ----------------------------------------------------------------------- :    -

                           0         *      2080         *      2100         *      2120         *
target                   : ----------------------------------------------------------------------- :    -
NbDRP2                   : AGGCAAATGGGCCTGATGTTGCAAAAGCACCCAATCTTGTGTTTAAAATAACGAGCCGGGTTCCATATAAA : 2001
Niben101Scf09472g02009.1 : AGGCAAATGGGCCTGATGTTGCAAAAGCACCCAATCTTGTATTTAAAATAACGAGCCGGGTTCCATATAAA : 2001
Niben101Scf06437g06008.2 : AGGCAAATGGGCCTGATGTTGCAAAAGCACCCAATCTTGTGTTTAAAATAACGAGCCGGGTTCCATATAAA : 2073
Niben101Scf13854g00010.1 : AGGATAGTGGAAATCTTGAAGCAAAA----TTCAATTCTGGTTGGGAAGGATA-------TTTCACAGAAG : 1837
Niben101Scf18730g00015.1 : AGGCAAATGGGTCTGATGGAGGAAAAGGACCTAGTCTCTTGTTCAAATTAACAAGCAGGGTTCAATACAAG : 1884
                           agg  a tgg   t  tg  g aaaa         t    tt   a   a         tt  a a aa

                                 2140         *      2160         *      2180         *      2200
target                   : ----------------------------------------------------------------------- :    -
NbDRP2                   : ACAGTTTTAAAGGCACACAGCGCTGTTATCTTGAAGGCCGAGAGTGTGGCAGATAAAATGAAGTGGTTAAG : 2072
Niben101Scf09472g02009.1 : ACAGTTTTAAAGGCACACAGCGCTGTTATCTTGAAGGCCGAGAGTGTGGCAGATAAAATGGAGTGGTTAAG : 2072
Niben101Scf06437g06008.2 : ACAGTTTTAAAGGCACACAGCGCTGTTATCTTGAAGGCCGAGAGTGTGGCAGATAAAATGGAGTGGTTAAG : 2144
Niben101Scf13854g00010.1 : TAATTCTT----GCCCAAAGTGCTGTTATTTTGAAGGCAGAAAGTTTGGCTGAGAAGAATGAATGGATAAA : 1904
Niben101Scf18730g00015.1 : ACTGTTCTTAAAGCCCAAAGTGCTGTTGTTTTGAAGGCAGAAAGTTTGGCTGAGAAGAATGAATGGATAAA : 1955
                               t  t    gc ca ag gctgtt t ttgaaggc ga agt tggc ga aa a   a tgg taa

                                   *      2220         *      2240         *      2260         *
target                   : ----------------------------------------------------------------------- :    -
NbDRP2                   : TAAATTGAGAACTGTCATCAGCTCTAAAGGCGGTCAAGTTAAGGGTGAATCTGGGCCACCTATCCGACATA : 2143
Niben101Scf09472g02009.1 : TAAATTGAGAACTGTCATCAGCTCTAAAGGCGGTCAAGTTAAGGGTGAATCTGGGCCACCTATCCGACATA : 2143
Niben101Scf06437g06008.2 : TAAATTGAGAACTGTCATCAGCTCTAAAGGCGGTCAAGTTAAGGGTGAATCTGGGCCACCTATCCGACATA : 2215
Niben101Scf13854g00010.1 : CAAATTGAAAAATGTCATAAGTTCTAAAGGAGGTCAGGTTATTGCTGATTCTGGTCCACCTGTGAGGCAAA : 1975
Niben101Scf18730g00015.1 : CAAATTGAAAAATGTCATAAGCTCTAAAGGAGGTCAGGTTATTGCTGATTCTGGTCCACCTGTGAGGCAAA : 2026
                            aaattga aa tgtcat ag tctaaagg ggtca gtta  g tga tctgg ccacct t  g ca a
```

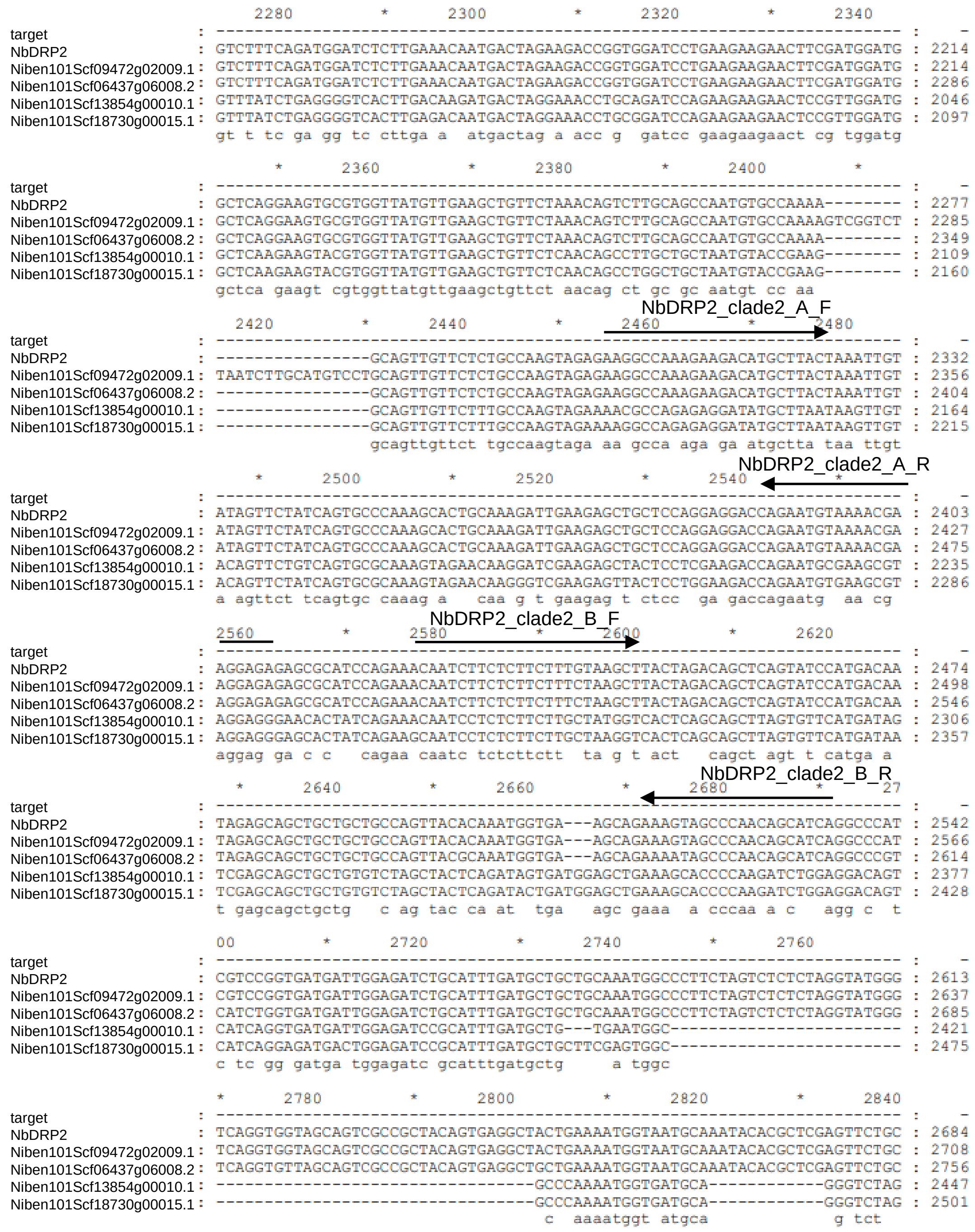

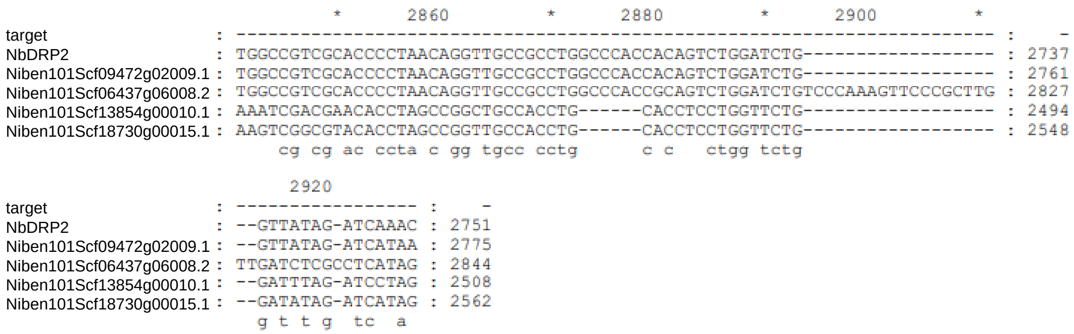

Supplementary Figure 2: The target sequence used for VIGS.
The VIGS target sequence inserted into the TRV vector is highlighted. The target sequence was determined in pssRNAit (https://www.zhaolab.org/pssRNAit/) using the sequence of Niben101Scf09472g02009.1. The nucleotide sequences of *NbDRP2* and its homologs were aligned using MUSCLE. Although the sequence in the boxed region in Niben101Scf06437g06008.1 is missing, the BLAST search using another *N. benthamiana* database, Nbe.v1 (Kurotani et al., 2023) revealed that the same sequence as Niben101Scf09472g02009.1 is present in this region. The primer sets used in this study are shown by arrows. A primer set 09472_F and 09472_R is used for the quantification of the expression of *NbDRP2* gene. Primer sets NbDRP2_clade2_A_F and NbDRP2_clade2_A_R, and NbDRP2_clade2_B_F and NbDRP2_clade2_B_R are used for the analysis of the expression of NbDRP2 homologs, Niben101Scf18730g00015.1 and Niben101Scf13854g00010.1.

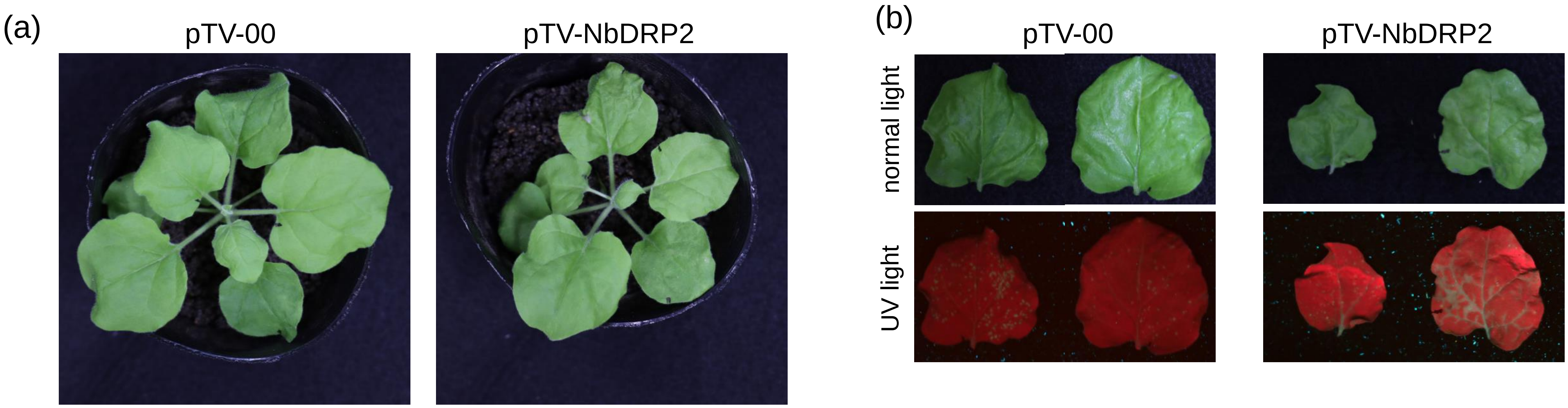

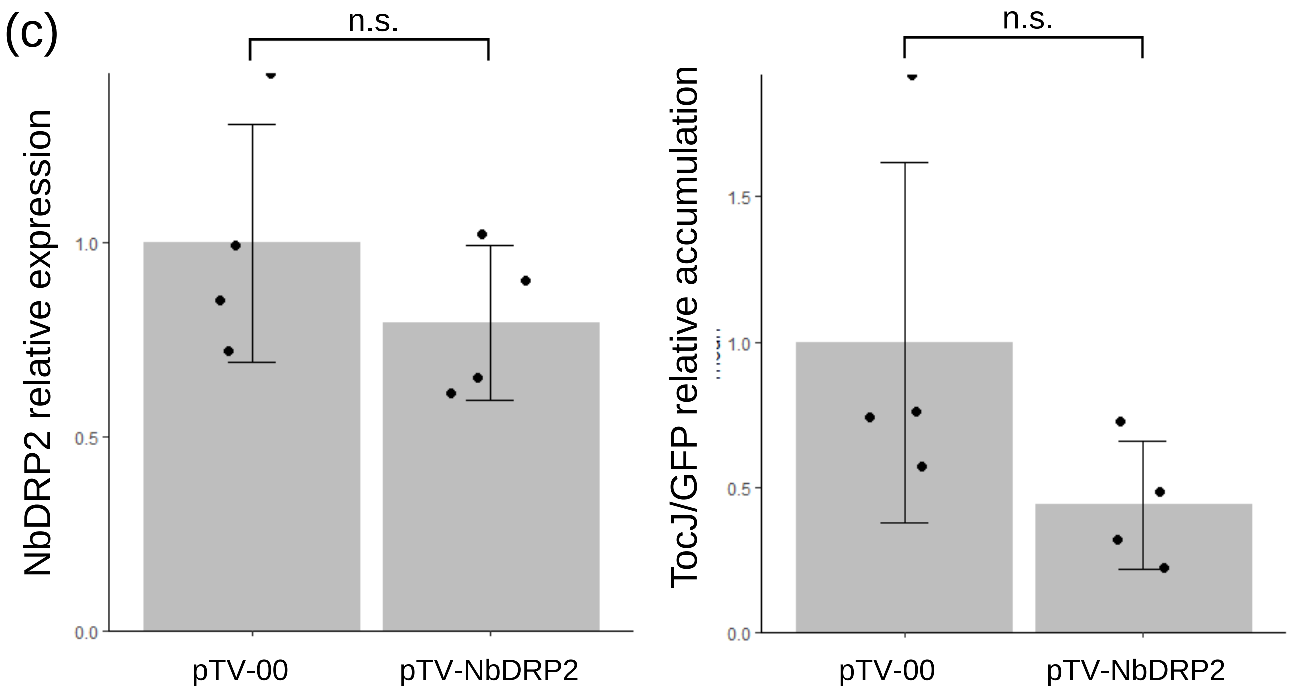

Supplementary Figure 3: The effect of the knockdown of *NbDRP2* on TocJ/GFP accumulation.

(a) Representative images of *Nicotiana benthamiana* plants inoculated with the TRV vector pTV-00 (empty vector) or pTV-NbDRP2. Images were taken at 10 days after TRV inoculation. (b) Representative images of TocJ/GFP inoculated leaves of the pTV-00- or pTV-NbDRP2-infected plants at 2 dpi. Two representative leaves are shown. (c) Quantification of *NbDRP2* gene expression and virus accumulation in TocJ/GFP inoculated leaves by RT-qPCR. The reference gene is *NbPP2A*. The mean value of *NbDRP2* gene expression and virus accumulation in the pTV-00 (control) group was set as 1. The graph represents the mean values and the error bars indicate the standard deviation of values for four biological replicates. n=4, n.s.: not significant by the Student's *t*-tests.

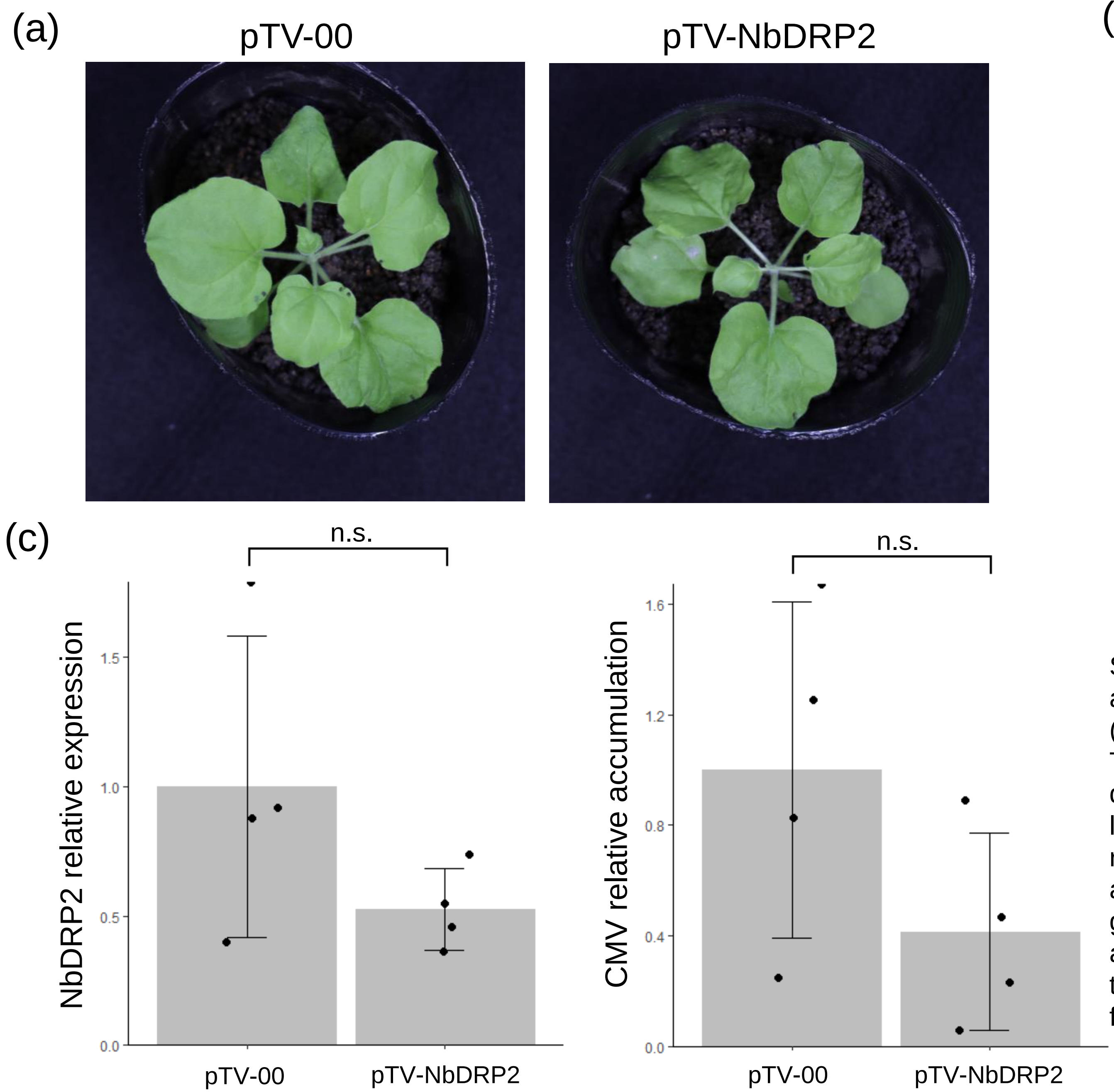

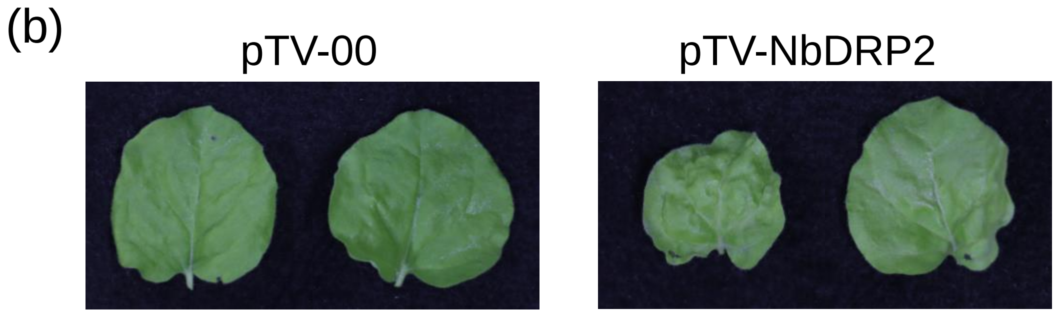

Supplementary Figure 4: The effect of the knockdown of *NbDRP2* on CMV accumulation.
(a) Representative images of *Nicotiana benthamiana* plants inoculated with the TRV vector pTV-00 (empty vector) or pTV-NbDRP2. Images were taken at 10 days after TRV inoculation. (b) Representative images of CMV inoculated leaves of the pTV-00- or pTV-NbDRP2-infected plants at 3 dpi. Two representative leaves are shown. (c) Quantification of *NbDRP2* gene expression and virus accumulation in CMV inoculated leaves by RT-qPCR. The reference gene is *NbPP2A*. The mean value of *NbDRP2* gene expression and virus accumulation in the pTV-00 (control) group was set as 1. The graph represents the mean values and the error bars indicate the standard deviation of values for four biological replicates. n=4, n.s.: not significant by the Student's *t*-tests.

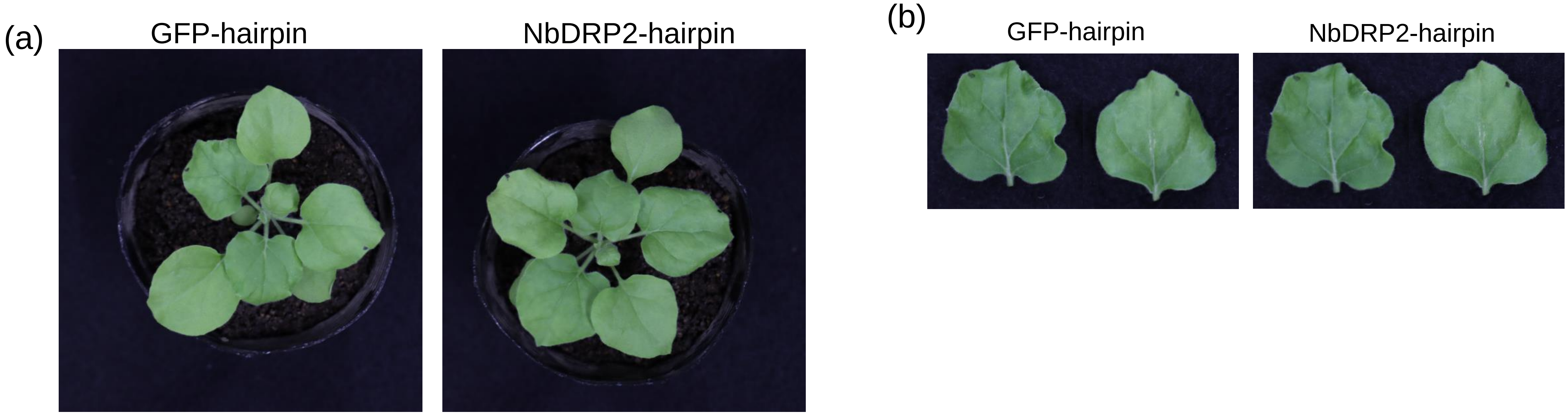

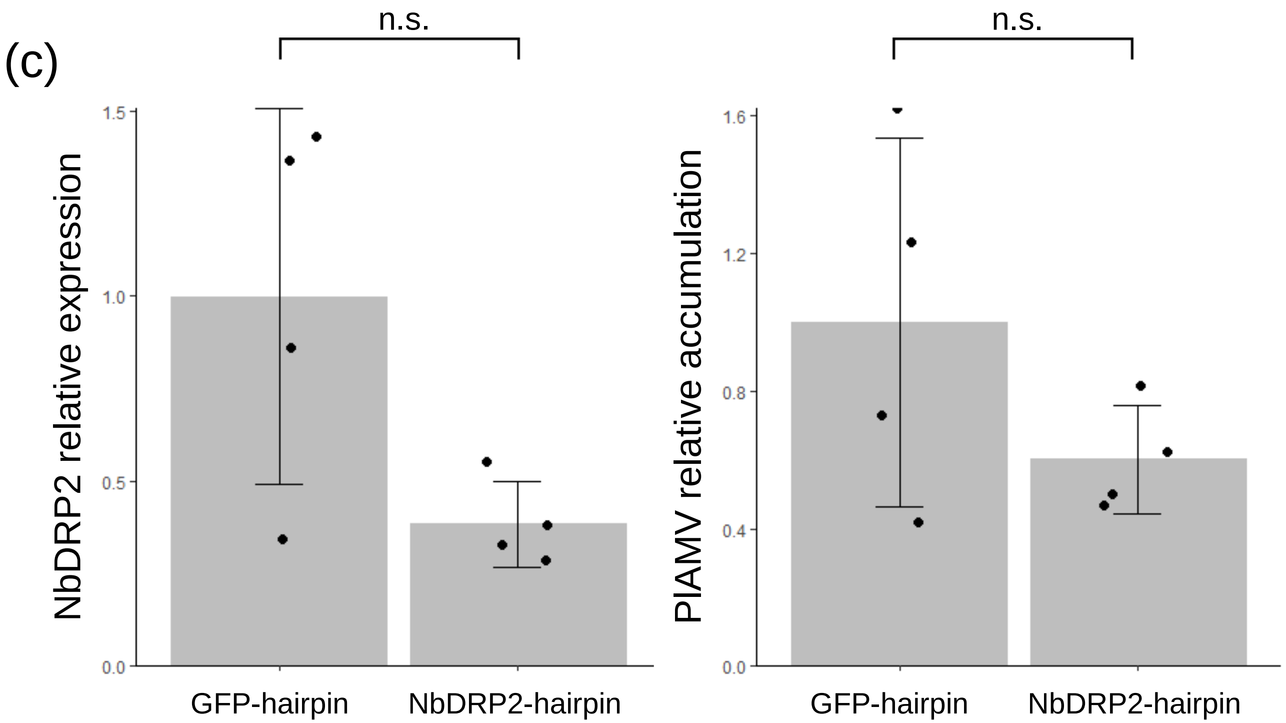

Supplementary Figure 5: The effect of transient knockdown of *NbDRP2* on PlAMV accumulation.

(a) Representative images of *Nicotiana benthamiana* plants expressing NbDRP2-hairpin or GFP- hairpin (control vector) by agroinfiltration. Images were taken at 2 days after agroinfiltration. (b) Representative images of PlAMV inoculated leaves of the NbDRP2-hairpin or GFP-hairpin-expressing plants at 2 dpi. Two representative leaves are shown. (c) Quantification of *NbDRP2* gene expression and virus accumulation in PlAMV inoculated leaves by RT-qPCR. The reference gene is *NbPP2A*. The mean value of *NbDRP2* gene expression and virus accumulation in the GFP-hairpin (control) group was set as 1. The graph represents the mean values and the error bars indicate the standard deviation of values for four biological replicates. n=4, n.s.: not significant by the Welch's *t*-tests.

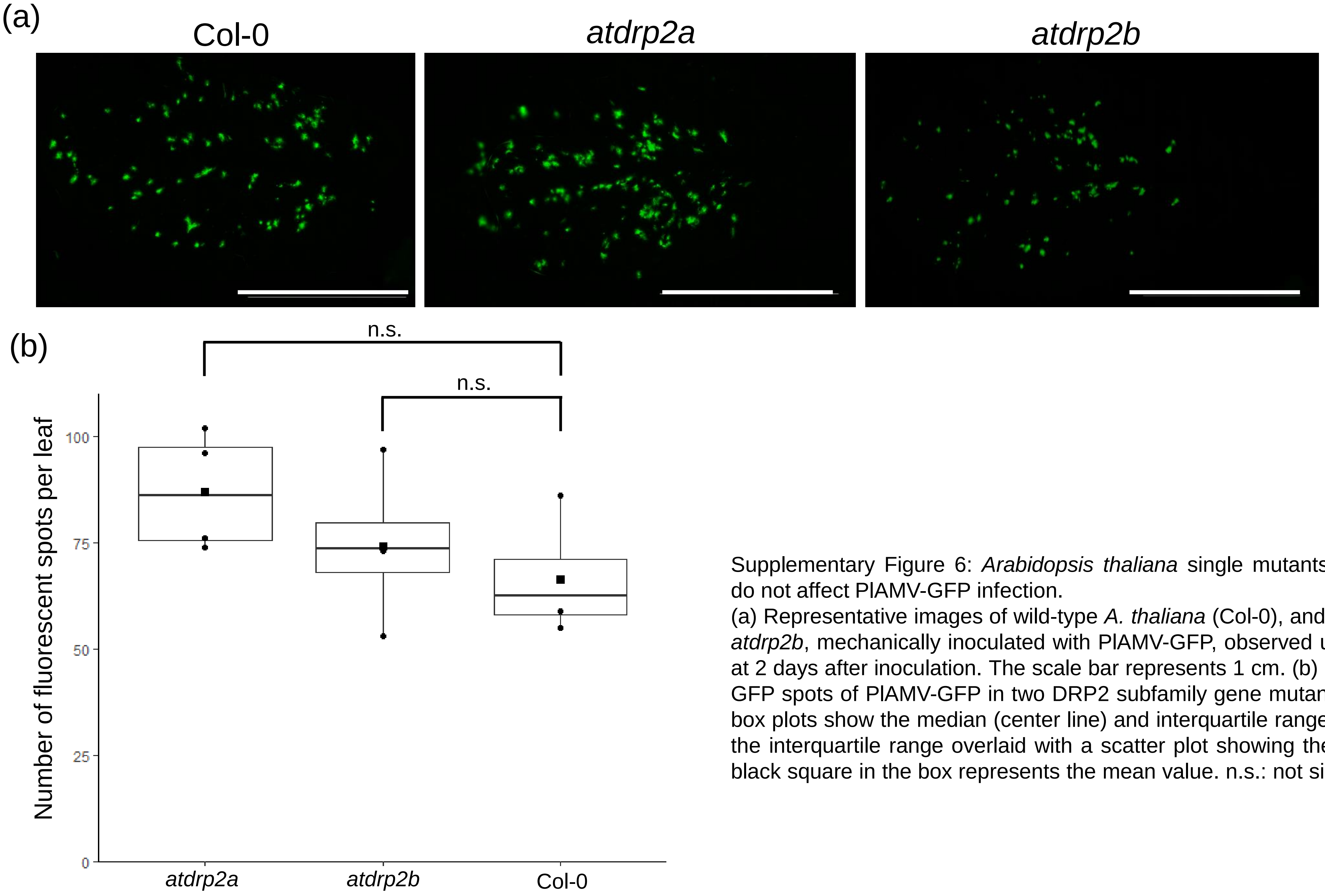

Supplementary Figure 6: *Arabidopsis thaliana* single mutants of the DRP2 subfamily gene do not affect PlAMV-GFP infection.

(a) Representative images of wild-type *A. thaliana* (Col-0), and its single mutants *atdrp2a* and *atdrp2b*, mechanically inoculated with PlAMV-GFP, observed using a fluorescent microscope at 2 days after inoculation. The scale bar represents 1 cm. (b) Quantification of the number of GFP spots of PlAMV-GFP in two DRP2 subfamily gene mutants and Col-0 using ImageJ. All box plots show the median (center line) and interquartile range, with whiskers up to 1.5 times the interquartile range overlaid with a scatter plot showing the values for each sample. The black square in the box represents the mean value. n.s.: not significant by the Student's *t*-test.

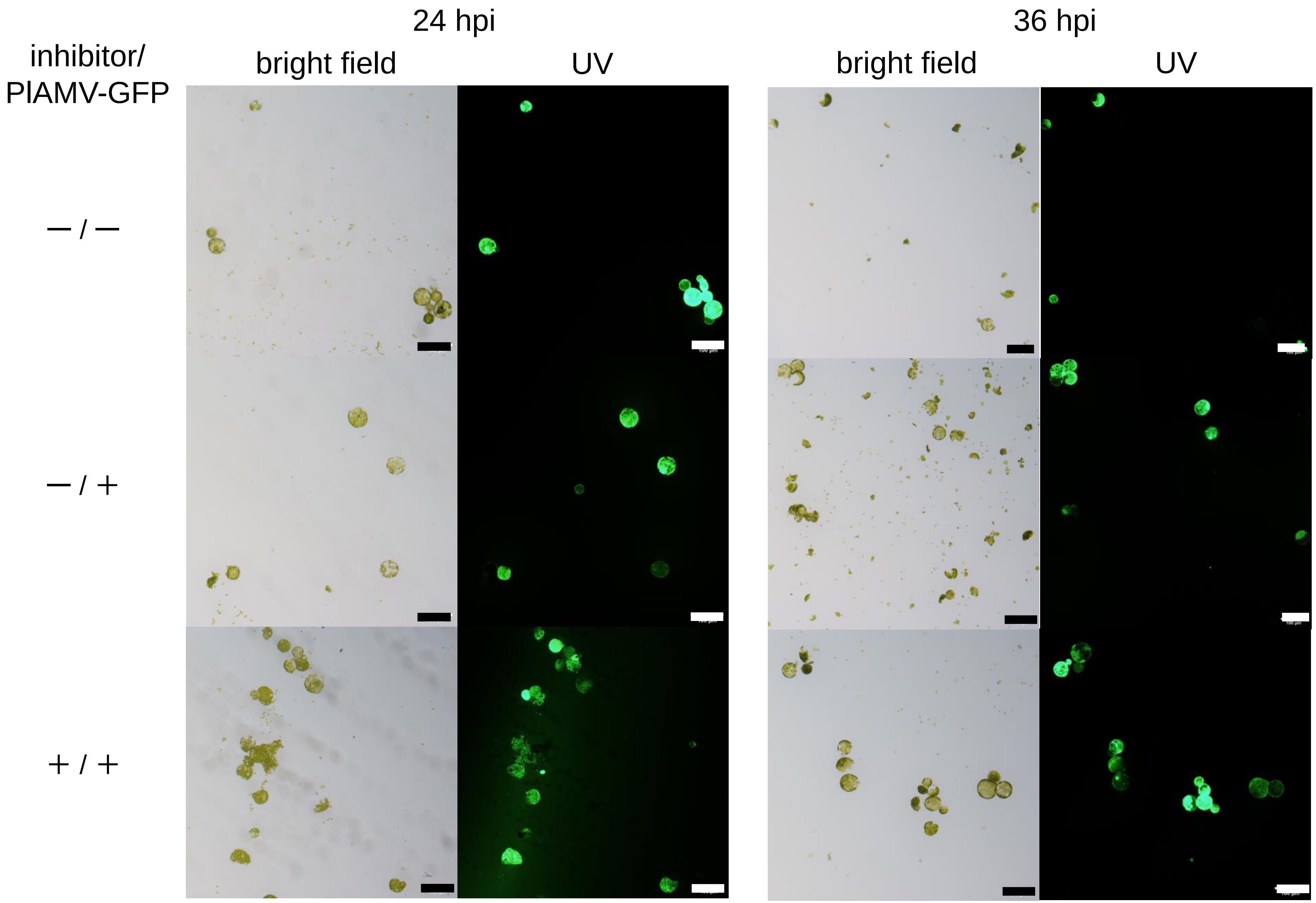

Supplementary Figure 7: Confirmation of viability of protoplasts treated with a dynamin inhibitor.
Protoplasts were stained with fluorescein diacetate to confirm the viability of protoplasts from each treatment after 24 and 36 hours of incubation.
The scale bar represents 100 µm.
